# Reverse Engineering of the Spindle Assembly Checkpoint

**DOI:** 10.1371/journal.pone.0006495

**Published:** 2009-08-04

**Authors:** Andreas Doncic, Eshel Ben-Jacob, Shmuel Einav, Naama Barkai

**Affiliations:** 1 Department of Molecular Genetics, Weizmann Institute of Science, Rehovot, Israel; 2 Department of Biomedical Engineering, The Iby and Aladar Fleischman Faculty of Engineering, Tel Aviv University, Ramat Aviv, Israel; 3 School of Physics and Astronomy, Beverly and Raymond Sackler Faculty of Exact Sciences, Tel Aviv University, Tel Aviv, Israel; 4 Department of Physics of Complex Systems, Weizmann Institute of Science, Rehovot, Israel; University of Glasgow, United Kingdom

## Abstract

The Spindle Assembly Checkpoint (SAC) is an intracellular mechanism that ensures proper chromosome segregation. By inhibiting Cdc20, a co-factor of the Anaphase Promoting Complex (APC), the checkpoint arrests the cell cycle until all chromosomes are properly attached to the mitotic spindle. Inhibition of Cdc20 is mediated by a conserved network of interacting proteins. The individual functions of these proteins are well characterized, but understanding of their integrated function is still rudimentary. We here describe our attempts to reverse-engineer the SAC network based on gene deletion phenotypes. We begun by formulating a general model of the SAC which enables us to predict the rate of chromosomal missegregation for any putative set of interactions between the SAC proteins. Next the missegregation rates of seven yeast strains are measured in response to the deletion of one or two checkpoint proteins. Finally, we searched for the set of interactions that correctly predicted the observed missegregation rates of all deletion mutants. Remarkably, although based on only seven phenotypes, the consistent network we obtained successfully reproduces many of the known properties of the SAC. Further insights provided by our analysis are discussed.

## Introduction

Proper chromosome segregation is a critical aspect of mitotic cell division; Failure in this process leads to aneuploidy, that often result in severe cellular defects, death or cancer [1]–[4]. The segregation process requires the polar attachment of the newly duplicated chromosomes to microtubules emanating from the two opposite poles. In budding yeast, the Spindle Pole Bodies (SPB), serve as the main microtubule organizing centers. In early S-phase, the SPB are duplicated and during metaphase the two SPB send out microtubules which attach to the chromosomes in a stochastic manner [5], [6]. More specifically, the microtubules attach to the kinetochore, a large multi-protein complex located on the centromere region of the chromosome [5]. Once all chromosomes are properly attached, meaning that precisely one of each duplicated chromosome is attached to each SPB, the chromosomes separate, with one set of chromosomes staying in the mother cell and the other set is pulled to the future daughter cell.

**Figure 1:**
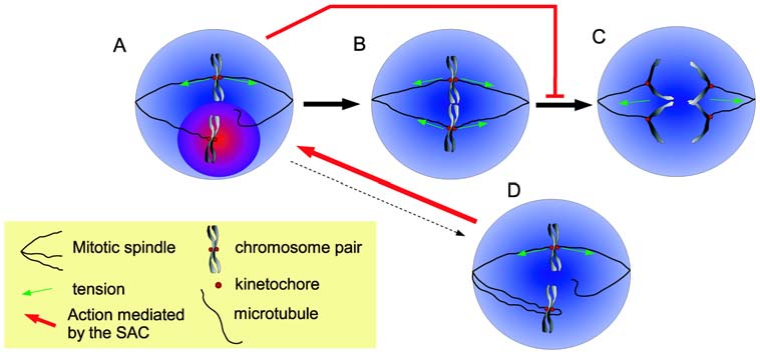
A schematic of the SAC. A. During metaphase, microtubules from the mitotic spindles stochastically search for the kinetochores (red dots) located on the chromosomes. Once microtubules have attached in a bipolar manner, tension (green arrows) is applied and the kinetochore-microtubule connection is stabilized. Meanwhile, the unattached kinetochore sends out a signal that stops anaphase commencement (red-blue gradient). B. After some variable time, all kinetochores are properly attached to the mitotic spindles and the “stop-anaphase” signal ceases. C. Anaphase commences rapidly after proper attachment. D. If a kinetochore pair is attached in a non-proper way (here a synthelic attachment is shown) the checkpoint detaches the faulty kinetochore-microtubule connection.

The Spindle Assembly Checkpoint (SAC) [7]–[9] is a control mechanism that safeguards the fidelity of this process (figure 1). First, if both sister chromosomes are erroneously attached to the SPBs, the SAC promotes microtubules detachment [10]. Second, the checkpoint arrests the cell cycle until all chromosomes are attached to the microtubules. To this end, any unattached kinetochore emits a diffusible signal that arrests the cell cycle.

The diffusible ‘stop-anaphase’ signal culminates in the inhibition of Cdc20, a vital activator of cell cycle progression [11]. Cdc20 is a cofactor of the Anaphase Promoting Complex (APC), a ubiquitin ligase that regulates many cell cycle processes [12]. When the APC is bound to Cdc20, the active APC^Cdc20^ complex degrades Pds1 (Securin), an event that triggers a cascade of reactions, leading ultimately to chromosome separation. Failure to inhibit Cdc20 therefore increases the probability of premature chromosome segregation leading to a quantifiable increase in the chromosomal missegregation rate [13]–[15].

The stop-anaphase signal originates at the unattached kinetochores where a set of highly conserved checkpoint proteins reside. Key proteins implicated in this process include Bub1, Bub3, Mad1, Mad2, Mad3 (BubR1) [16], [17], Mps1 [18], [19] and Ipl1 (Aurora B) [20]. These proteins interact on the kinetochore, forming inhibitory complexes that diffuse away to inhibit Cdc20. Inhibition of Cdc20 occurs in two primary ways: First, the inhibitor complexes bind Cdc20 and prevents it from binding the APC (sequestration) [21]. Second, Cdc20 degradation is enhanced [22]. It is known that Mad2 sequesters Cdc20 whereas the Mitotic Checkpoint Complex (MCC) composed of Mad2, Mad3 and Bub3 both sequesters and degrades Cdc20 [23], [24]. Other ‘Mad -Bub’ complexes such as Bub3-Mad3 and Mad2-Mad3 might also be formed and/or participate in the inhibition of Cdc20. Additional mechanisms involved in Cdc20 inhibition might also include Cdc20 and Pds1 phosphorylation [25]–[27].

Detailed genetic and biochemical studies revealed a great deal of information about the interactions between the SAC proteins and the means by which Cdc20 is inhibited. In addition, recent theoretical work began addressing some aspects of their integrated functions. In a previous study, we described a general interplay between the strength of Cdc20 inhibition and the rate of checkpoint inactivation. Based on this analysis, we argued that models in which Cdc20 is inhibited at the kinetochore itself are inconsistent with the relevant spatial (spindle size) and temporal (cell-cycle timing) constraints. Rather, the results called for a model in which Cdc20 is inhibited by a diffusible inhibitor that is generated on the kinetochore [28], [29]. More recently, Sear and Howard [30] devised the first model for the SAC in metazoan cells considering the further implication of the large metazoan cells on SAC performance. This model was later extended by Mistry et al [31], to include also the Aurora B interaction on the SAC as well as the kinetochore-microtubule interactions. The effect of different Mad2 conformers in metazoan cells was further analyzed by Ibrahim et al [32], [33] and Simonetta et al [34].

Our previous work focused on the essential properties of the SAC, but did not attempt to capture the full details of the network. Here, we attempted to proceed beyond this general description and examine the possibility of deducing the detailed interactions between the checkpoint proteins using the quantitative phenotype of gene deletion mutants. To this end, we began by formulating a general model that enables predictions of chromosome missegregation rate for any given set of interactions between the SAC proteins. Following our previous analysis, this model relies on the generation of diffusible Cdc20 inhibitors from the kinetochores. Our general model allows us to screen over many different putative SAC networks, corresponding to different assumptions about which molecular species participate in the inhibitory complexes, which proteins facilitate the formation of these complexes on the kinetochore, and the means by which these complexes inhibit Cdc20.

In the second stage of the analysis, we measure the chromosome loss rates of seven yeast mutant strains, each deleted of one or two of the key protein components of the SAC. Finally, we screen for networks that are consistent with these values. We find that we these seven phenotypes are sufficient to tightly constrain the possible models. The predicted network reproduces many of the known features of the SAC and provides new insights about the function of this checkpoint.

We view our study as only one of the initial steps towards devising formal approaches for reverse engineering of biological systems in general, and the SAC in particular. Therefore, before describing the approach details, we would like to draw the attention to some of its limitations. First, our approach although comprehensive, did require us to make some simplifications and assumptions about the behavior of this system. At present, experimental evidence is not sufficient to justify or refute some of these assumptions. For example, we only look at the system in steady state and thus do not capture any of the dynamical interactions needed to assemble the SAC proteins on the kinetochores and to initiate the checkpoint. In reality it is likely that recruitment to the kinetochore does involve non-linear interactions. Regulatory feedbacks are also hard to rule out. Similarly, we assumed that chromosome missegragation rate is proportional to the level of APC^Cdc20^. While it is highly likely that these two are indeed correlated in a monotonic fashion, it is also plausible that the relationship is non-linear. The number of free parameters over which we screened was rather large, and we compared them to only seven quantitative phenotypes that were derived to a limited resolution. In addition, some parameters not screened over were fixed by literature values, which are again, known only to some limit.

It is interesting that despite these inevitable limitations, the reverse engineering theme was quite successful in pinpointing the key features of the checkpoint. This make us optimistic regarding further developments in this direction.

## Results

### Overview of our reverse-engineering approach

Extensive genetic and molecular studies have revealed the core protein components of the SAC and described many of the interactions between them. In this work, we asked whether it is possible to “reverse engineer” the network topology using information about the quantitative phenotypic effects of deleting individual protein components. Specifically, we wished to define the active SAC (in steady-state during Metaphase) by the following: Characterizing the interactions between the different network proteins during their activation on the kinetochore and defining the means by which the activating complexes inhibit Cdc20 through sequestering or degradation. As a quantitative phenotype, we considered the Chromosome Missegregation Rate (CMR). Since the main function of the checkpoint is to prevent chromosome missegregation events, deletion of any SAC protein is expected to affect the missegregation rate in a manner related to the specific role of this protein in the checkpoint. The CMR can thus provide a link between a quantitative, observable phenotype and the molecular interactions within the SAC network.

**Figure 2:**
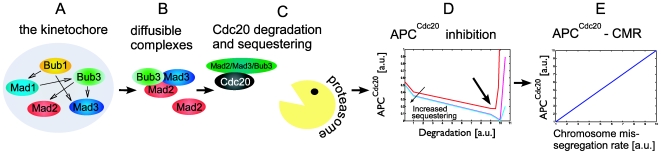
An overview of the SAC model. The main function of the stalling part of the SAC is to prevent premature activation of APC^Cdc20^. Failure to do so results in premature anaphase entry which leads to chromosome missegregation. A general model connecting the phenotype (chromosome missegregation) with the interactions of the SAC proteins on the kinetochore and in the cytoplasm was formulated. A–B All SAC proteins assemble on the unattached kinetochores, interact, and promote the creation of activated diffusible factors (proteins and complexes) composed of Mad2, Mad3 and Bub3. C These factors inhibit the Cdc20 by degradation and sequestering. The relative contribution of each SAC-protein to the sequestering and degradation rates is determined. D Cooperation between Cdc20 sequestering and degradation minimizes the APC^Cdc20^ (arrow) and determines the actual level of the inhibition rates. E Further, a quantitative relation between the APC^Cdc20^ and the CMR is assumed. Hence the SAC topology is connected to the phenotype. Knowing the CMR rate and the rate for all single SAC-deletions it is possible to compare any given putative topology with the real CMR values and thus search for solutions whose behavior is consistent with the observed phenotype.

Our reverse-engineering strategy is shown schematically in Figure 2. As a starting point, we formulated a general model that describes the interactions between the SAC proteins during their activation on the kinetochore and in the cytoplasm. We assume that the kinetochore-associated activation of the SAC proteins culminates in the generation of active molecular species/factors (active protein or an active complex) that diffuse to the cytoplasm to inhibit Cdc20. Each of the kinetochore-bound SAC proteins (Mad1, Mad2, Mad3, Bub1 and Bub3) can promote the association and binding of any of the other proteins to the kinetochore. Outside factors such as Ipl1 and Mps1 can also promote kinetochore association. This activation of factor “A” by factor “B” (resulting for example from promoting kinetochore association or complex formation) is quantified by a single parameter. Notably, by choosing different parameters (and by assigning a certain subset of parameters to zero) this general model can describe distinct network topologies. Hence, the output of this first part is a set of molecular species (single proteins or complexes) capable of inhibiting Cdc20, each produced at a certain rate and released to diffuse in the cytoplasm.

Next we assumed that each of the inhibitory factors can potentially both sequester Cdc20 and promote its degradation. The relative contribution of each factor to these reactions is quantified, again, by a single parameter. Hence, given some specific network topology, this modeling framework provides us with a quantitative estimate of the degree by which each of the SAC-protein contributes to the total sequestering and degradation rates. It should be emphasized that the model only gives us the overall contribution of each protein in the context of some specific network. It doesn't say anything about the timescales in the system or the relative fractions of proteins binding each other.

Notably, while the model defines the relative contribution of each protein to the total sequestering and degradation rates, the actual value of these rates still needs to be determined, as does their effect on the level of APC^cdc20^. To determine this, we first solved a simplified model of the interactions between Cdc20 and the APC and the inhibitory complex(es). This simplified model does not consider the detailed formation of the inhibitory complexes, but summarizes the network function by two parameters: the rate of Cdc20 sequestration, and the rate of Cdc20 degradation. With the exception of these two parameters, all other parameters of this model were defined based on available data. As we show below, we find that optimal performance (minimal level of active APC^Cdc20^) is obtained for some optimal values of the sequestration and degradation rates. We assumed that the wild-type network complies with these optimal levels, thus minimizing the level of APC^Cdc20^. Finally, the CMR was assumed to be linearly proportional to the level of active APC^Cdc20^. Taken together, this framework allowed us to predict, for any given set of putative SAC interactions, how gene deletions or other perturbations would affect the CMR.

With this model at hand, we proceeded to measure CMR in mutants deleted of the SAC-proteins. Using these measured rates as a template, we performed a computational screen to define the set of parameters (or network topologies) which properly explain the deletion phenotypes. Below we provide more details about this procedure and discuss its results.

### A generic model for the SAC

#### The SAC-proteins interact on the kinetochores to form inhibiting factors that diffuse to sequester and degrade Cdc20

SAC signaling originates on the unattached kinetochores, where all SAC core proteins (Bub1, Bub3, Mad1, Mad2 and Mad3) assemble, interact and promote the creation of the diffusible factors (individual proteins and protein complexes) that inhibit the Cdc20 during metaphase. Here we describe a model which determines, for a given network topology, the relative contribution of each SAC protein to the rates by which Cdc20 is degraded or sequestered.

**Figure 3:**
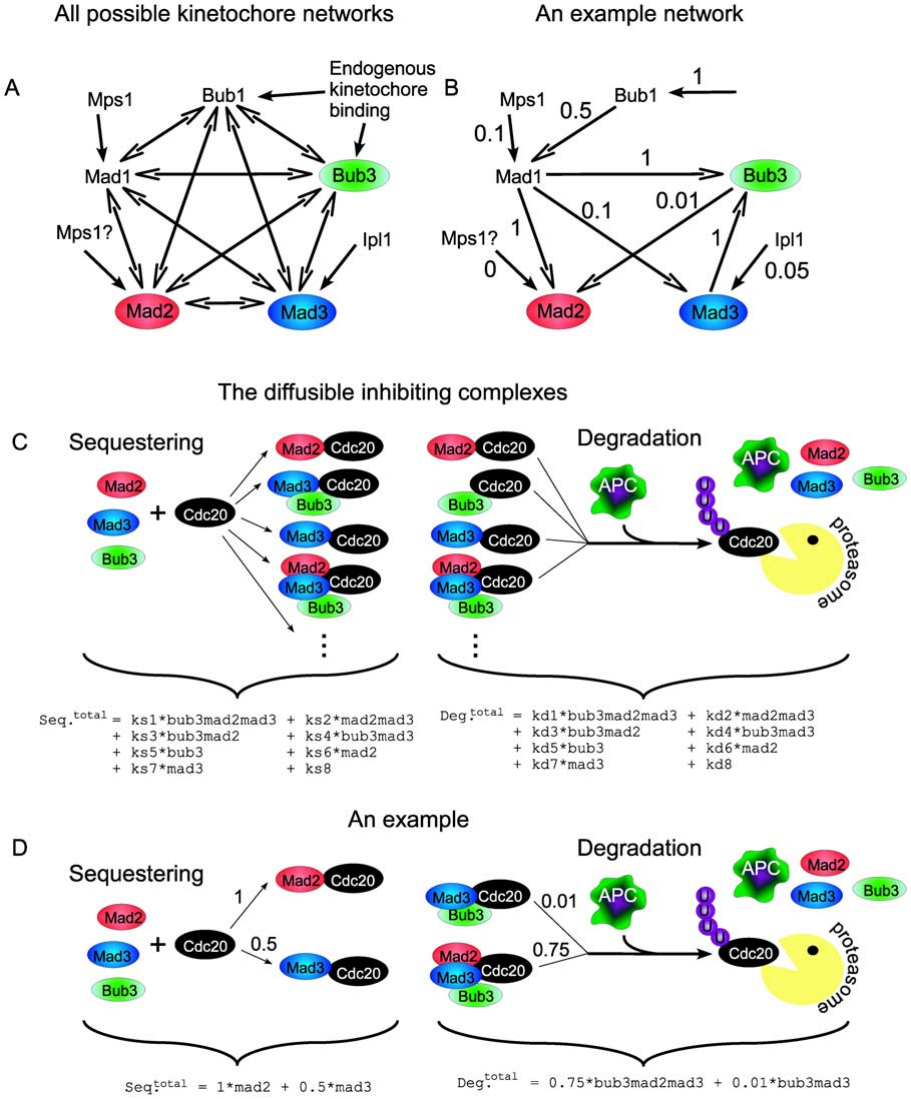
The SAC-proteins promote Cdc20 sequestering and degradation. A. A model representing the interactions of the SAC core proteins on the kinetochores was formulated. Each SAC protein was represented by a node and each node was connected to five edges. The edges represented possible activations from the four other SAC proteins or from some external source. The ten edges connecting the SAC proteins were all assigned a direction and a value between 0 and 1, representing the relative strength of the interaction. The five external activations were only assigned a value. An interaction whose value is set to zero does not exist. Hence by randomizing the interaction directions and their weights our model can capture a vast number of different kinetochore interaction networks. In the end the relative activity of Mad2, Mad3 and Bub3 was obtained. B. An example kinetochore interaction network. C. Mad2, Mad3 and Bub3, whose relative activity level was determined by the kinetochore interactions, can inhibit Cdc20, either by forming complexes or by themselves. Each activated factor (protein/complex) is assigned two values: one for its relative sequestering strength and one for its relative strength of degradation. Again, the values varied between 0 and 1. The relative degree of sequestration and degradation for each factor was calculated as the product of the kinetochore activities of all its components multiplied by the specific sequestration/degradation rate for this factor and normalized with the ‘total’ sequestering/degradation (see Equations 2–6). It is known that Bub3 alone does not promote Cdc20 inhibition [51] and that Mad2 alone does not degrade Cdc20 [52] hence we exclude these activated proteins from the computational screen. D. An example set of sequestering and degrading proteins and complexes: for simplicity, the constant contributions are omitted here.

Our generic kinetochore model consists of five nodes, each representing one of the five SAC proteins. Five possible edges are attached to each node: four edges connecting it to the other checkpoint proteins, and one additional edge for potential outside interactions (e.g. inputs from Ipl1/Mps1, see below and figure 3A). Each edge in the network is assigned a value between 0 and 1. The value of the edge, say, from Mad1 to Mad2 describes the (relative) strength by which Mad1 “activates” Mad2 on the kinetochore. An edge of strength “zero” corresponds to a non-existing interaction (see section 1 and 7.2 in supporting information S1 for more details about how the weights were chosen). Since the kinetochore serves as a scaffold for the SAC proteins, we assume that all edges are unidirectional, i.e. A can recruit B, or B can recruit A, but both recruitments are not possible.

Note that we are only interested in the general wiring of the network but do not attempt to capture the nature of the activation. The output of this kinetochore network is the relative levels of activated Mad2, Mad3 and Bub3. This means that the kinetochore network here represents a fully loaded kinetochore which stays loaded until anaphase commences. The activation strengths thus represent the overall contributions from/to each protein to a final time-invariant state. It is very likely that the dynamical assembling of the kinetochores is a much more complex process [31]. Here, however, we only look at the already assembled kinetochores (see figure 3 and section 1 in supporting information S1).

As an illustration, we show in Figure 3B one particular example of a putative kinetochore network. For this specific case, the activated levels of the different proteins are calculated as follows:
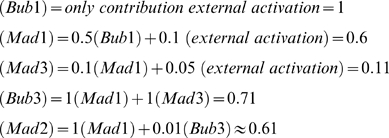
(1)


In this example, the kinetochore activates Mad2, Mad3 and Bub3 with relative strengths of 0.61∶0.11∶0.71, so that Bub3 is the potentially strongest Cdc20 inhibitor. Note that Bub1, the most upstream component in this example, is activated by some undefined external factor.

Next, we consider the inhibition of Cdc20 by the diffusible inhibitors generated by the network above. We consider the three proteins known to participate in this inhibition: Mad2, Mad3 and Bub3, as well as all possible complexes that can be formed between them totaling to seven inhibitory factors (figure 3C). The relative contribution of each inhibiting factor to the sequestering and/or degradation rates is calculated as the product of the ‘activity ‘of each of its components, as described above.

These activities are additionally multiplied by some specific sequestering and degradation constant which ranges from zero (when the complex cannot sequester or degrade Cdc20) to one (strong sequestering or degradation). The relative strength of the sequestering and degradation for each inhibitor were chosen in the same way as for the edges in the kinetochore network (see above and section 1 and 7.2 in supporting information S1). Note that the activity of Bub3, Mad2 and Mad3 will be determined partly from the kinetochore and partly from the activity of the sequestering and degrading complexes. This implies that, when varying all rates, there will inevitably be redundant solutions. We choose to keep this formalism for two reasons. First, the prediction of zero activation (no interaction), for some specific interaction, is non-redundant. Second, the effect of the checkpoint proteins which are not directly involved in the sequestration/inhibition (Bub1 and Mad1) can only be captured through the kinetochore network.

The total sequestering and degradation rates are given by the sums of all these contributions, with an additional constant term representing sequestering/degradation mediated by other sources, e.g. Cdc20 phosphorylation (see the supporting information S1 for a discussion about the choice of these constants). Together, we obtain the overall sequestration and degradation rates (*Seq^tot^* and *Deg^tot^*, respectively):
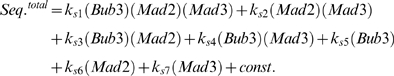
(2)

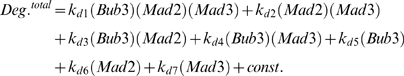
(3)


The different rates, ‘*k’* are again determined as part of our screen. As a putative example, consider the network in Figure 3B, whose kinetochore activation is described in Equation 1, and consider the case where Cdc20 sequestration is mediated only by Mad2 and Mad3 (individually), with relative weights of 1 and 0.5, whereas degradation is carried out by Bub3-Mad3 and by Mad2-Mad3-Bub3 with relative weights of 0.01 and 0.75 (figure 3D). In this case the total sequestering and degradation rates are as follows:
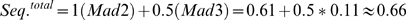
(4)

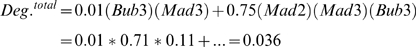
(5)


Using this, we can define the relative contribution of each protein or protein complex to the sequestering and degradation as follow:
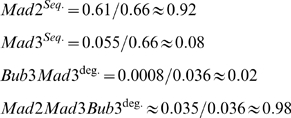
(6)


Thus, for these putative parameter values, Mad2 is the main sequestering agent and the Mad2Mad3Bub3 complex is the main degrading agent. Deleting Mad2, for instance, will result in ∼92% reduction in the sequestering rate and ∼98% reduction in the degradation. Deletion of Mad3, on the other hand, will result in only ∼8% reduction in sequestration rate, but ∼98% reduction in the degradation rate.

Note that in our actual simulations we first screen to find the best sequestration and degradation rates and then determine the relative contributions of the SAC proteins as in the example above. Hence we cannot say with any certainty that we use the actual values of the total degradation and sequestration (see below). The expression in Eq. 5 is only important in describing the relative contribution of each protein to the total rates, to enable the calculations of deletion mutant phenotypes.

#### By combining sequestering and degradation the SAC promotes maximal inhibition of APC^Cdc20^

The model discussed above provides the relative contribution of each SAC-protein to the general sequestering and degradation rates. Next, we had two objectives: First we wished to determine the actual values of these rates. Second, we wished to understand how Cdc20 sequestering together with Cdc20 degradation affects the amount of active APC^Cdc20^.

**Figure 4:**
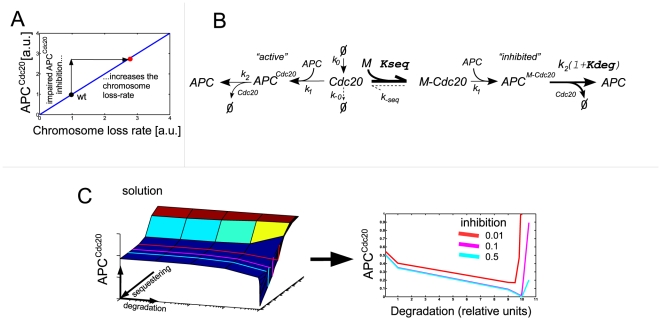
Interplay between sequestering and degradation ensures minimal APC^Cdc20^. A. A general model for the Cdc20 sequestering and degradation interplay was formulated. The action of the diffusible SAC-proteins/complexes is generalized as a diffusible inhibitor ‘*M*’. Hence Cdc20 is being spontaneously generated at some rate *k_0_* and can either bind a free APC, forming active APC^Cdc20^ or be sequestered forming M-Cdc20. The sequestered Cdc20 can also bind APC forming an inactive APC^MCdc20^. Both Cdc20 and MCdc20 are degraded by the APC. The degradation of the inhibited complex is enhanced by the checkpoint (*k_deg_>k_2_*). For simplicity it was assumed that APC binds free and sequestered Cdc20 at the same rate (*k_1_*). In order to make the model general it was also assumed that Cdc20 can degrade in an APC independent manner (*k_-0_*) and that Cdc20 can desequester (*k_-seq_*). Both these rates are small compared to the sequestering and degradation rates. B. The relation between the sequestering, the degradation and the APC^Cdc20^ level: The level of APC^Cdc20^ varies with the sequestration and degradation rates. We note that for any sequestering rate there is a degradation rate for which the APC^Cdc20^ inhibition is optimal. See the right panel for the relation between APC^Cdc20^ and the degradation rate for three different fixed sequestration rates (units are molecules^−1^s^−1^). This holds true for all cases where inhibition is good (APC^Cdc20^/APC^total^<0.01). We conclude that the wild type sequestering and degradation rates are such that they minimize the APC^Cdc20^. The reason behind this optimization of the inhibition is that the degradation regulates the balance between the amount of free inhibiting complexes, Cdc20 and APC. C. The level of APC^Cdc20^ drives the chromosomal missegregation rate: An impaired ability to inhibit APC^Cdc20^ is translated into an increase in the chromosomal missegregation rate (see main text and supporting information S1 for details).

To this end, we used a simplifying modeling approach to examine how the different modes of Cdc20 combine to limit the level of APC^Cdc20^. We formulated a generalized model that captures the inhibition reaction, and the association of Cdc20 with the APC. Cdc20 can either bind the APC (forming active APC^Cdc20^-complex), or be sequestered by some diffusible inhibitory factor “*M*” (giving rise to a sequestered Cdc20, “*MCdc20*”), (figure 4A). The sequestered Cdc20 can also bind the APC forming inactive APC^MCdc20^. Further we assumed that both APC^Cdc20^ and APC^MCdc20^ can degrade Cdc20. The model was formulated using the following set of differential equations:
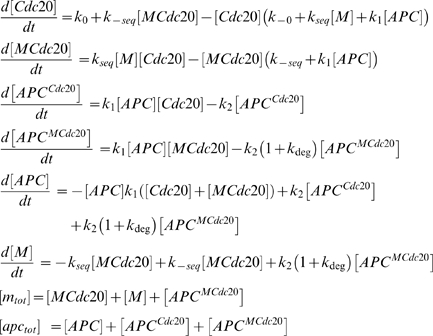
(7)


With the exception of the Cdc20 inhibition rates (*k_deg_* and *k_seq_*), all other rates were fixed as follows: Cdc20 production rate (*k_0_*) was estimated based on reports of Cdc20 half-life and copy–number, while the APC-independent degradation of Cdc20 (*k_-0_*) and its spontaneous desequestering rates (*k_-seq_*) were assumed to be negligible. The association rate of the APC-Cdc20 complex (*k_1_*), as well as the non-APC dependent degradation rate (*k_2_*) had a minor effect on the results and were arbitrarily fixed (see section 2 in supporting information S1 for details about all rates).

We solved for the level of APC^Cdc20^ as a function of the Cdc20 inhibition rates *k_deg_* and *k_seq_*. Surprisingly, a non-monotonic relation between the APC^Cdc20^ level and *k_deg_* was observed. Upon increasing *k_deg_* with a fixed *k_seq_*, the APC^Cdc20^ levels initially decreases, as expected, but then exhibit a rapid increase (figure 4B). This last increase reflects the fact that APC is, in effect, sequestered by the MCdc20 complex. Increasing Cdc20 degradation lowers the amount of the MCdc20 complex and consequently results in the increase of APC. Above some limiting value, this increase in APC can compensate for the decrease in Cdc20, resulting in an *increase* in the level of active the APC^cdc20^ compex. To proceed, we assumed that the checkpoint is optimized, so that in the wild-type, *k_deg_* and *k_seq_* are tuned with each other to ensure minimal levels of APC^Ccd20^. Note that this optimality relation gives a direct relationship between the degradation and sequestration rates, but still does not predict their actual values. During the screen (see below) we found that only one of these sets of rates can accurately describe the observed phenotype of the deletion mutants. It should also be noted that all the optimal rates used in the screen fulfill our previously stated constraints on the SAC (strong APC^Cdc20^ inhibition, rapid reactivation and resistance to noise in the Cdc20 production) [28], [29] (see section 2.4 in supporting information S1 for more details).

#### The APC^Cdc20^ level is proportional to the chromosome missegregation rate

Up to now we describe how we predict APC^20^ levels for a given set of parameters both in wild-type cells (as a function of the inhibition parameters) and in cells that are deleted in various checkpoint proteins (as a function of the interactions between them). The last point in our approach is to connect the level of APC^cdc20^ to the measurable phenotype, namely the rate of chromosomal missegragation. It is likely that CMR is monotonic with the APC^Cdc20^, but the exact functional form is not known. For simplicity, we assume that the proportionality is direct (see section 3 in supporting information S1 for details about this assumption).

### Computational screen

Our general model described above depends on a large number of parameters. Different sets of parameters correspond to different networks with different properties. Each putative (wild-type) network is assumed to be optimal, defining a chromosome missegregation rate of one. As explained above, the model now provides us with the ability to predict the impact from deletions of any network component (protein) on the CMR for any network topology. This is done by setting the rates associated with this protein to zero and calculating the effect of the deletion on the APC^Cdc20^. Assuming that the APC^Cdc20^ is directly proportional to the CMR, we get the following relation for the CMR of some arbitrary protein *A*:
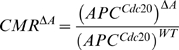
(8)


Hence, to try and identify the realization that best corresponds to the actual checkpoint in yeast, we searched for networks that properly predict deletion phenotypes (chromosome missegregation rates) of strains deleted of key checkpoint proteins.

We determined experimentally the chromosome loss rates of cells deleted of Bub1, Bub3, Mad1, Mad2 and Mad3. This was done using the ALF-faker assay [35] (see table 1). We also deleted Sgo1 and measured the associated increase in the chromosomal loss rate in order to determine how large a fraction of the measured Bub1 and Bub3 loss rates stems from the detachment part of the network (see sections 4–6 in supporting information S1 for details). The reason for not using published data of CMR [36] is that the available data is not complete and uses a different, noisier assay [37].

**Table 1:** See supporting information S1 for a comparison with previous measurements of chromosome missegregation and for an explanation about the rescaling of the Bub1, Bub3 and Mad1 rates.

deletion	X-fold increase in missegregation rate	Standard deviation	Rescaled increase	Rescaled standard deviation
***bub1***	34.7	5.3	1.8	0.28
***bub3***	21.6	3.0	1.4	0.19
***mad1***	2.6	0.29	-	-
***mad2***	2.7	0.27	-	-
***mad3***	1.4	0.15	-	-
***sgo1***	24.1	3.7	-	-
***mad1mad3***	1.8	0.27	-	-
***mad2mad3***	1.8	0.22	-	-

**Figure 5:**
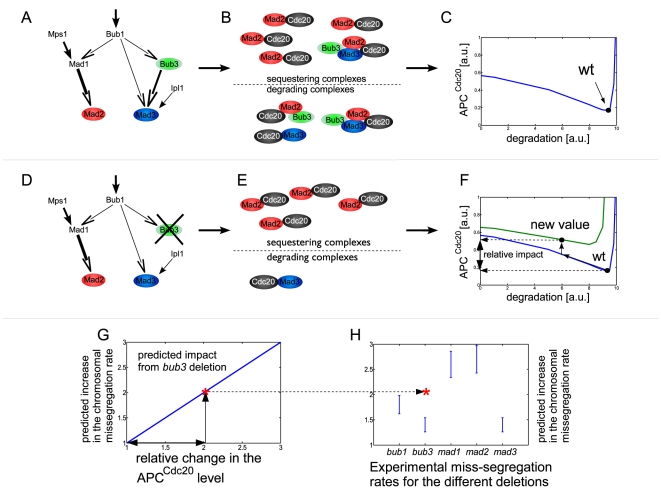
The screen. A. A kinetochore network is chosen and its weights and the contribution from external sources (here denoted ‘Ipl1’ and ‘Mps1’, see main text for details) are randomized. B. One or several activated factors (proteins/complexes) composed of Bub3 Mad2 and Mad3 contribute in a varying degree to the sequestering and degradation rates. Next, the relative contribution of each factor to the total Cdc20 sequestering and/or degradation was calculated. Note that the same complex can contribute to both sequestering and degradation (here MCC does that). C. The total sequestration and degradation rates from all factors are normalized so that they correspond to rates giving an optimal (minimal) level of APC^Cdc20^. Here the relationship between the APC^Cdc20^ and the degradation rate is shown for a fixed sequestration rate. D–E. Once the wild-type network was defined we proceeded to sequentially delete all the SAC-proteins. The impact of deleting Bub3 is shown here. On the kinetochore Mad3 is less activated and all complexes containing Bub3 also disappears. F. The decrease of the sequestering and degradation is translated into a new APC^Cdc20^ level. This decrease is represented here by a shift on the x-axis (degradation) and from the blue curve (stronger sequestering) to the green curve (weaker sequestering). G. The relative impact on the APC^Cdc20^ is than translated into a predicted change in the chromosomal missegregation rate for this network and this mutation and compared (H.) with the experimental values. The *bub3* deletion here does not fall within the accepted range thus disqualifying this particular topology. For details about the screen see the supporting information S1.

We screened through approximately thirty million possible networks by comparing them with the experimentally measured values of the single deletion mutants. In the screen we considered all possible kinetochore networks and inhibiting factors by varying the kinetochore edge configurations, their activation values as well as the number of sequestering and degradation complexes and their weights. The sequestration rate for the putative network was also varied. The latter determined the location of the wild type optimum and was found to be constant for all solutions (see Figure 5 and section 7 in supporting information S1 for details).

This screen identified 105 consistent networks, which correctly predicted all of the five deletion phenotypes within 5% of the experimental values. These 105 consistent networks represented 24 different kinetochore-interactions graphs. Next, the networks were clustered so that all redundant solutions were removed (see figure 5, and section 7.3 in supporting information S1 for details) giving us 82 networks representing 20 different topologies (see also section 7.3 in supporting information S1).

#### Distinguishing between consistent networks based on the phenotype of double-deletion mutants

**Figure 6:**
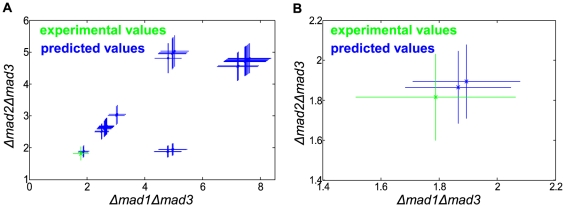
Experimental versus predicted values for the double deletions. A. The experimental (green) versus the predicted (blue) values. The x- and y-axis represent chromosome missegregation rates for the *mad1mad3* and the *mad2mad3* double deletions whereas the lines extending from each point represent one standard deviation. B. A zoom-in on the lower left corner of A. The rightmost (worse) predicted value is not likely to represent the real topology since it contains many highly unlikely interactions (see main text for details).

Each of the consistent networks from the screen was used to predict the impact of the ten possible double deletions. To further differentiate between the consistent solutions we choose to test our predictions for the *mad1mad*3 and *mad2mad3* double deletions which best differentiated between the models. These double deletion strains were constructed and tested for the rate of chromosome missegregation (see figure 6 and table 1). Notably, we predicted and observed a strong buffering effect for both these mutants since the missergregation rate is by far lower than the product of the CMR for the individual deletions of *mad1mad3* and *mad2mad3*.

Of the 82 possible checkpoint networks only two accurately predicted the chromosome missegregation rates for the two double mutants. One of those appeared less plausible since it relied on highly improbable interactions and complexes. More precisely, it requires the existence of a Mad2-Bub3 complex and interaction between Mad1 and Mad3. No such complex or interaction is known to exist. We also note that the only reason this solution came out of the analysis is the slight experimental difference between the *mad1* and *mad2* deletions (table 1). This difference could however, be due to experimental noise. To ensure against the possibility that we missed some alternative (redundant) solutions in the screen we analyzed the resulting network and showed that no such redundancy existed (see section 8 in supporting information S1 for details).

**Figure 7:**
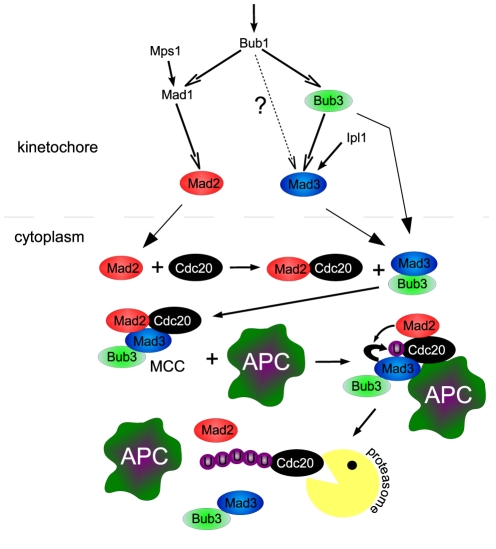
A proposed mechanism for the SAC. On the kinetochore, Bub1 is activated either endogenously or by Ipl1 or some other factor. Once in place, Bub1 together with Mps1 promotes Mad1 activation which in turn activates Mad2. Bub1 also activates Bub3 that, together with Ipl1, activates Mad3. Subsequently, the active Mad2 diffuses and sequesters Cdc20. The resulting Mad2-Cdc20 complex then binds to the activated Bub3-Mad3 complex and forms the MCC. The MCC proceeds to bind the APC where the Cdc20 gets ubiquinated and degraded. The degradation of the Cdc20 recycles the other MCC components which restarts the process.

The network that emerged from the screen and validation as being most consistent with the phenotypes of the deletion mutants is shown in figure 7. In this network, Bub3 is activated by Bub1, Mad1 is activated by an external factor and Bub1. Bub3 and some other external factor activate Mad3 and Mad1 activates Mad2. The Mad2 and the MCC (Mad2-Mad3-Bub3) sequester the Cdc20 and Bub3Mad3 and the MCC degrade it. Unlike the Mad2 and MCC functions, which are known, the role of Bub3Mad3 as a Cdc20 degrading complex was not established yet.

Two additional aspects of the solution should be noted. First, the possibility that Bub1 activates Mad3 on the kinetochore cannot be ruled out with this method since Bub3 and Mad3 always acts together in the final consistent topology (this topology was clustered with the consistent one). We believe, however, that this is an unlikely connection because Mad3 needs Bub3 (which needs Bub1) for kinetochore localization [38], [39] and because Bub1 and Mad3 are most likely spatially distant on the kinetochore [40]. Second, we see a relative large contribution to the sequestering and degradation that comes from the constant term in equations 2 and 3, (see section 9 in supporting information S1). This might reflect inhibition of APC^Cdc20^ through Cdc20 and Pds1 phosphorylation. Alternatively, it could result from an experimental inaccuracy (see section 9 in supporting information S1 for more details)

## Discussion

A working SAC is crucial for chromosomal integrity and impairments to its function leads to an increase in the chromosome missegregation rate. We presented an initial approach to reverse engineering the system. The final outcome of our numerical and experimental analysis is a qualitative description of the structure and function of the cell cycle stalling part of the spindle assembly checkpoint. We now discuss some of the results in relation to existing literature.

### The kinetochore and the diffusible inhibitors

Consistent with previous reports [38], [41], we found that Bub1 is the most upstream component on the kinetochore. The nature of the Bub1 activation is not known; It could be endogenous, promoted by Ipl1 [42], or induced by some other factor. Although proposed [43], we could not identify a solution with Bub3 as the most upstream factor. Below Bub1 the kinetochore network bifurcates into two branches, consistent with previous suggestions [7]. In the first branch, Bub1, together with some external factor, activates (or recruits) Mad1 which, in turn, activates (or recruits) Mad2. In the other branch, Bub1 activates Bub3 who, together with some external factor, activate Mad3. It is likely that the external activations of Mad1 and Mad3 reflect the actions of Mps1 and Ipl1, which were shown to be necessary for their respective functions [44], [45]. Notably, all these interactions were previously reported [21], [38], [39], [44]–[46] yet we made no assumptions about their existence.

The consistent network further predicts that Mad2 and MCC sequester Cdc20, whereas Bub3-Mad3 and MCC degrade it. These results suggest that the separation of the checkpoint into two branches also reflect a functional division: The Mad2-branch promotes Mad2 activation and consequently Cdc20 sequestration, whereas the Bub3/Mad3 branch induces the formation of the Bub3-Mad3 complex which promotes Cdc20 degradation. Our analysis suggests that the MCC is formed in the cytoplasm by combining Bub3-Mad3 with Mad2-Cdc20. Bub3-Mad3 thus promotes Cdc20 degradation by forming the MCC which is necessary for the Cdc20 ubiquitination by the APC. However, our model does not exclude the possibility that, Bub3-Mad3 degrade Cdc20 in the absence of Mad2 since Mad3 does bind Cdc20 weakly even in the absence of Mad2 [47]. Interestingly, such a mechanism, whereby Bub3Mad3 degrade Cdc20 independently of Mad2, was recently suggested [48] (see section 9 in supporting information S1 for more details).

The idea that the MCC is produced by the binding of Mad2-Cdc20 and Bub3-Mad3 was suggested before [7] and is supported by the fact that Mad2 and Cdc20 (forming the Mad2-Cdc20 complex) show similar kinetochore kinetics as Bub3 and Mad3 [40]. Interestingly, the formation of MCC as a secondary complex in the cytoplasm might explain the enigmatic existence of MCC in non-mitotic cells [49], [50]. These MCC could be formed long after the inactivation of the last kinetochore by ‘leftover’ Mad2-Cdc20 and Bub3-Mad3. Moreover, these ‘non-mitotic’ MCC would then have to compete with other non-mitotic substrates to get access to the APC which might prolong their halftime. It is also possible that the Cdc20 degradation depends on some other Metaphase specific event such as Cdc20 phosphorylation which might increase the MCC longevity in non-mitotic cells.

### Sequestering and degradation

Our model of Cdc20 kinetics predicts that the cooperation between Cdc20 sequestering and degradation creates an optimized inhibition by minimizing the level of the APC^Cdc20^. The fact that the sequestering and degradation machinery themselves are combined into one network makes perfect sense since it ensures that the coordination between the two modes of inhibition remains intact.

Our analysis also predicts that increasing the degradation rate is far more deleterious than down-regulation of either the sequestering or the degradation rate. In support of this, over-expression of Mad3 (a key degrading protein) leads to a higher CMR than deleting either Mad3 or Mad2 [36]. Another prediction is that certain combinations of double deletions are buffered. The reason for this is the non-linear dependence of APC^Cdc20^ on the sequestering and degradation rates. We verified this predicted buffering experimentally for both the *mad1mad3* and the *mad2mad3* double deletion strains.

### In conclusion

The SAC is a sophisticated network composed of many different and partially overlapping functions. In this work, we analyzed one of the checkpoint functions: the ability of unattached kinetochore to arrest the cell cycle. Our analysis is by no means complete but, hopefully, provides some insight into the interrelationship between the different protein components and the different mechanisms for Cdc20 inhibition.

## Materials and Methods

### The A-Like Faker (ALF) assay

The mating tester BE287α (*ura4 car2 gal2*), the wild type reference strain (BY4742) and the deletion strains (BY4742 background) were grown overnight in YPD medium at 30°C. The cells were then diluted and re-grown to exponential phase. After this, 1 OD unit of the wt reference- and the deletion-strain (∼2.5e7 cells) were mixed with 3OD units of BE287α, the cell were spun down and the supernatant discarded. Before the OD measurement of the BE287α they were sonicated at 3 W for 2×30 s. The cells were then resuspended in 100 µl sterile double distilled water and placed on a 0.45 µm filter on a YPD plate and incubated at 30°C for four hours for mating. After this the cells were collected from the filter and plated on SC ura^−^ plates. The colonies were then counted approximately 40 h after plating. The chromosome missegregation rates were then calculated as a function of the wild type loss rate and the deletion strain loss rate (see supporting information S1 for a detailed description)

The set of ODEs describing the impact of sequestering and degradation on the APC^Cdc20^ was solved with a custom built Ringe-Kutta algorithm using MATLAB, also the numerical screen was performed with custom built software using MATLAB.

## Supporting Information

Supporting Information S1 Supporting Information.
(0.81 MB DOC)

## References

[1] Homer HA, McDougall A, Levasseur M, Yallop K, Murdoch AP (2005). Mad2 prevents aneuploidy and premature proteolysis of cyclin B and securin during meiosis I in mouse oocytes.. Genes Dev.

[2] Shonn MA, McCarroll R, Murray AW (2000). Requirement of the spindle checkpoint for proper chromosome segregation in budding yeast meiosis.. Science.

[3] Rajagopalan H, Lengauer C (2004). Aneuploidy and cancer.. Nature.

[4] Kops GJ, Weaver BA, Cleveland DW (2005). On the road to cancer: aneuploidy and the mitotic checkpoint.. Nat Rev Cancer.

[5] Westermann S, Drubin DG, Barnes G (2007). Structures and functions of yeast kinetochore complexes.. Annu Rev Biochem.

[6] McAinsh AD, Tytell JD, Sorger PK (2003). Structure, function, and regulation of budding yeast kinetochores.. Annu Rev Cell Dev Biol.

[7] Musacchio A, Salmon ED (2007). The spindle-assembly checkpoint in space and time.. Nat Rev Mol Cell Biol.

[8] Cleveland DW, Mao Y, Sullivan KF (2003). Centromeres and kinetochores: from epigenetics to mitotic checkpoint signaling.. Cell.

[9] Lew DJ, Burke DJ (2003). The spindle assembly and spindle position checkpoints.. Annu Rev Genet.

[10] Ruchaud S, Carmena M, Earnshaw WC (2007). Chromosomal passengers: conducting cell division.. Nat Rev Mol Cell Biol.

[11] Hwang LH, Lau LF, Smith DL, Mistrot CA, Hardwick KG (1998). Budding yeast Cdc20: a target of the spindle checkpoint.. Science.

[12] Peters JM (2006). The anaphase promoting complex/cyclosome: a machine designed to destroy.. Nat Rev Mol Cell Biol.

[13] Visintin R, Prinz S, Amon A (1997). CDC20 and CDH1: a family of substrate-specific activators of APC-dependent proteolysis.. Science.

[14] Uhlmann F, Lottspeich F, Nasmyth K (1999). Sister-chromatid separation at anaphase onset is promoted by cleavage of the cohesin subunit Scc1.. Nature.

[15] Haering CH, Farcas AM, Arumugam P, Metson J, Nasmyth K (2008). The cohesin ring concatenates sister DNA molecules.. Nature.

[16] Li R, Murray AW (1991). Feedback control of mitosis in budding yeast.. Cell.

[17] Hoyt MA, Totis L, Roberts BT (1991). S. cerevisiae genes required for cell cycle arrest in response to loss of microtubule function.. Cell.

[18] Weiss E, Winey M (1996). The Saccharomyces cerevisiae spindle pole body duplication gene MPS1 is part of a mitotic checkpoint.. J Cell Biol.

[19] Hardwick KG, Weiss E, Luca FC, Winey M, Murray AW (1996). Activation of the budding yeast spindle assembly checkpoint without mitotic spindle disruption.. Science.

[20] Chan CS, Botstein D (1993). Isolation and characterization of chromosome-gain and increase-in-ploidy mutants in yeast.. Genetics.

[21] De Antoni A, Pearson CG, Cimini D, Canman JC, Sala V (2005). The Mad1/Mad2 complex as a template for Mad2 activation in the spindle assembly checkpoint.. Curr Biol.

[22] Pan J, Chen RH (2004). Spindle checkpoint regulates Cdc20p stability in Saccharomyces cerevisiae.. Genes Dev.

[23] Sudakin V, Chan GK, Yen TJ (2001). Checkpoint inhibition of the APC/C in HeLa cells is mediated by a complex of BUBR1, BUB3, CDC20, and MAD2.. J Cell Biol.

[24] Hardwick KG, Johnston RC, Smith DL, Murray AW (2000). MAD3 encodes a novel component of the spindle checkpoint which interacts with Bub3p, Cdc20p, and Mad2p.. J Cell Biol.

[25] Holt LJ, Krutchinsky AN, Morgan DO (2008). Positive feedback sharpens the anaphase switch.. Nature.

[26] Tang Z, Shu H, Oncel D, Chen S, Yu H (2004). Phosphorylation of Cdc20 by Bub1 provides a catalytic mechanism for APC/C inhibition by the spindle checkpoint.. Mol Cell.

[27] Chung E, Chen RH (2003). Phosphorylation of Cdc20 is required for its inhibition by the spindle checkpoint.. Nat Cell Biol.

[28] Doncic A, Ben-Jacob E, Barkai N (2006). Noise resistance in the spindle assembly checkpoint.. Mol Syst Biol.

[29] Doncic A, Ben-Jacob E, Barkai N (2005). Evaluating putative mechanisms of the mitotic spindle checkpoint.. Proc Natl Acad Sci U S A.

[30] Sear RP, Howard M (2006). Modeling dual pathways for the metazoan spindle assembly checkpoint.. Proc Natl Acad Sci U S A.

[31] Mistry HB, MacCallum DE, Jackson RC, Chaplain MA, Davidson FA (2008). Modeling the temporal evolution of the spindle assembly checkpoint and role of Aurora B kinase.. Proc Natl Acad Sci U S A.

[32] Ibrahim B, Diekmann S, Schmitt E, Dittrich P (2008). In-silico modeling of the mitotic spindle assembly checkpoint.. PLoS ONE.

[33] Ibrahim B, Dittrich P, Diekmann S, Schmitt E (2008). Mad2 binding is not sufficient for complete Cdc20 sequestering in mitotic transition control (an in silico study).. Biophys Chem.

[34] Simonetta M, Manzoni R, Mosca R, Mapelli M, Massimiliano L (2009). The influence of catalysis on mad2 activation dynamics.. PLoS Biol.

[35] Yuen KW, Warren CD, Chen O, Kwok T, Hieter P (2007). Systematic genome instability screens in yeast and their potential relevance to cancer.. Proc Natl Acad Sci U S A.

[36] Warren CD, Brady DM, Johnston RC, Hanna JS, Hardwick KG (2002). Distinct chromosome segregation roles for spindle checkpoint proteins.. Mol Biol Cell.

[37] Hieter P, Mann C, Snyder M, Davis RW (1985). Mitotic stability of yeast chromosomes: a colony color assay that measures nondisjunction and chromosome loss.. Cell.

[38] Kerscher O, Crotti LB, Basrai MA (2003). Recognizing chromosomes in trouble: association of the spindle checkpoint protein Bub3p with altered kinetochores and a unique defective centromere.. Mol Cell Biol.

[39] Millband DN, Hardwick KG (2002). Fission yeast Mad3p is required for Mad2p to inhibit the anaphase-promoting complex and localizes to kinetochores in a Bub1p-, Bub3p-, and Mph1p-dependent manner.. Mol Cell Biol.

[40] Howell BJ, Moree B, Farrar EM, Stewart S, Fang G (2004). Spindle checkpoint protein dynamics at kinetochores in living cells.. Curr Biol.

[41] Farr KA, Hoyt MA (1998). Bub1p kinase activates the Saccharomyces cerevisiae spindle assembly checkpoint.. Mol Cell Biol.

[42] Vigneron S, Prieto S, Bernis C, Labbe JC, Castro A (2004). Kinetochore localization of spindle checkpoint proteins: who controls whom?. Mol Biol Cell.

[43] Gillett ES, Espelin CW, Sorger PK (2004). Spindle checkpoint proteins and chromosome-microtubule attachment in budding yeast.. J Cell Biol.

[44] King EM, Rachidi N, Morrice N, Hardwick KG, Stark MJ (2007). Ipl1p-dependent phosphorylation of Mad3p is required for the spindle checkpoint response to lack of tension at kinetochores.. Genes Dev.

[45] Hardwick KG, Murray AW (1995). Mad1p, a phosphoprotein component of the spindle assembly checkpoint in budding yeast.. J Cell Biol.

[46] Burke DJ, Stukenberg PT (2008). Linking kinetochore-microtubule binding to the spindle checkpoint.. Dev Cell.

[47] Burton JL, Solomon MJ (2007). Mad3p, a pseudosubstrate inhibitor of APCCdc20 in the spindle assembly checkpoint.. Genes Dev.

[48] Nilsson J, Yekezare M, Minshull J, Pines J (2008). The APC/C maintains the spindle assembly checkpoint by targeting Cdc20 for destruction.. Nat Cell Biol.

[49] Fraschini R, Beretta A, Sironi L, Musacchio A, Lucchini G (2001). Bub3 interaction with Mad2, Mad3 and Cdc20 is mediated by WD40 repeats and does not require intact kinetochores.. Embo J.

[50] Poddar A, Stukenberg PT, Burke DJ (2005). Two complexes of spindle checkpoint proteins containing Cdc20 and Mad2 assemble during mitosis independently of the kinetochore in Saccharomyces cerevisiae.. Eukaryot Cell.

[51] Larsen NA, Al-Bassam J, Wei RR, Harrison SC (2007). Structural analysis of Bub3 interactions in the mitotic spindle checkpoint.. Proc Natl Acad Sci U S A.

[52] King EM, van der Sar SJ, Hardwick KG (2007). Mad3 KEN boxes mediate both Cdc20 and Mad3 turnover, and are critical for the spindle checkpoint.. PLoS ONE.

