# Supplementary material for: Reverse Engineering of the Spindle Assembly Checkpoint
Source: PLoS One. 2009 Aug 4;4(8):e6495. doi: 10.1371/journal.pone.0006495 (PMC2714964; doi:10.1371/journal.pone.0006495)
Supplement: Supporting Information S1 — Supporting Information. (0.81 MB DOC) [file pone.0006495.s001.doc]

**1 The construction of the kinetochore networks**

We assumed that any of the five core SAC proteins (Bub1, Bub3, Mad1, Mad2 and Mad3) could interact with each other on the kinetochore. With "interaction" we mean activation/recruitment to the kinetochore by either some kind of unspecified physical interaction or by phosphorylation. We do not assume anything particular about the nature of the specific interactions. Hence we can only predict that "A activates B" but no know *how* the activation is done. This gave us a total of ten possible interactions among the five core SAC-proteins (not counting external activations). Each such interaction could go in either direction or be absent. This gave us a total possible of 3^10^ or 59049 possible networks (figure 3 and figure S1A). Worth noting is the fact that we only consider positive interactions. The reason for this is simple: the kinetochore activates the "stop-anaphase" signal on a scaffold. It wouldn’t make much sense to evolve a mechanism that specifically bind proteins to a scaffold in order to inhibit other proteins there when it is easier to "inhibit" them by not binding them to the scaffold in the first place. Also, no evidence of any kind on inhibiting interaction on the kinetochore among the SAC proteins exists to the best of our knowledge.

Next we constructed an algorithm that recursively searched each network and discarded any feedback loops found. The reason behind this assumption is that this model only considers the final state of the kinetochore where all SAC proteins are already assembled. It is both possible and likely that there are several feedback loops when the SAC proteins assemble on the kinetochore, yet once in place, the final contribution from some protein A to some other protein B should be constant. Conceptually one might consider a model where the kinetochore gets ‘more/less’ activated during metaphase which would correspond to some positive/negative feedback loop in the interaction network. This possibility is not supported by any experimental evidence so we choose to disregard it. In the end we got 29281 different kinetochore networks which were later used in the screens.


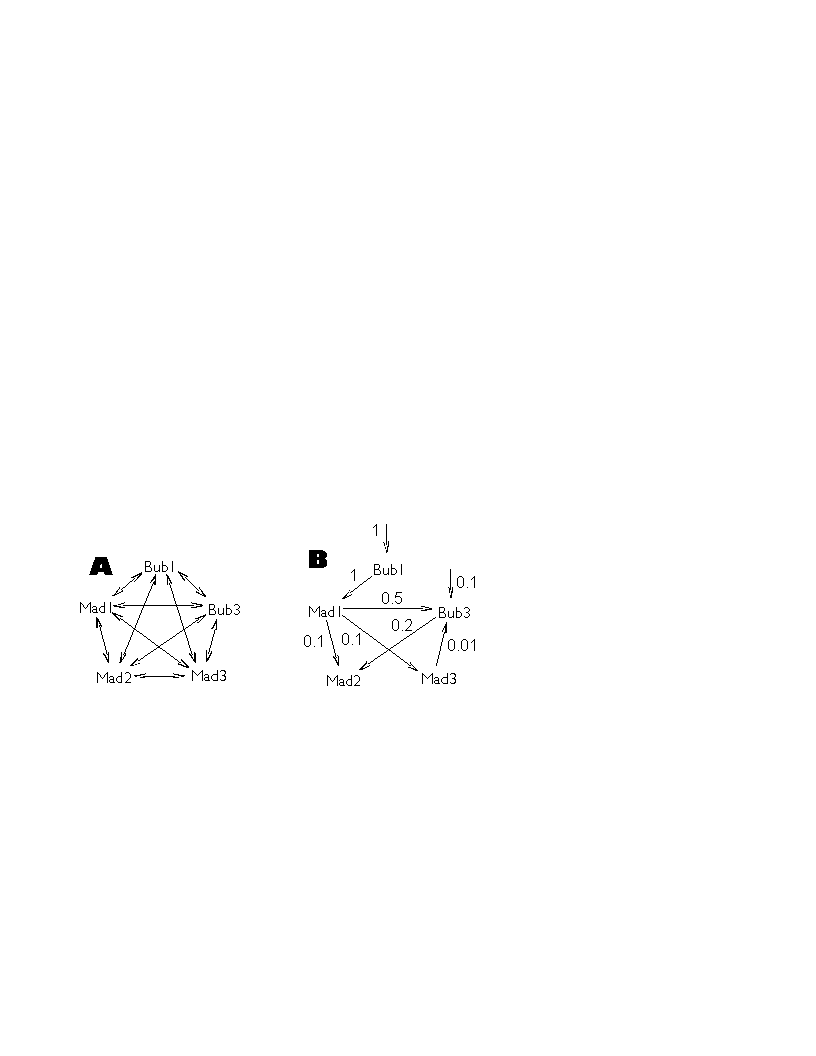


**Figure S1: (A)** The possible interactions on the kinetochore. Note that each node (SAC-protein) can also have some external activation. (B) An example network with weights and external activations.

The interactions between the nodes are assumed to be linear and the numbers used are only describing the relative degree of activation between the proteins for a final steady-state, in order to establish the impact of different kinetochore configurations on the chromosome missegregation rate. In the example from figure 1B, we get the following relative activities:

(1)

Note that the numbers here are with very high precision i.e. “0.22002” rather than 0.22 or 0.2. The reason for this is to illustrate how the calculation was done rather than imply that such small differences are important (see also below in section 7.2 for a discussion about how the weights were chosen).

By going over all possible kinetochore networks varying the interaction strengths it is likely that several networks will be redundant in the sense that two different network (by topology or rates, see figure S10) will produce the same outcome. Here we chose to go over all networks and then cluster the redundant solutions to get the unique solutions (see section 7 below).

**2 The overall model: How the sequestering/degradation affect the APC^Cdc20^ inhibition**

Following our previous results [1,2] a model for the interaction between the APC, Cdc20 and some generalized diffusible inhibitor ‘*M*’ was formulated. We assumed that the Cdc20 is being produced at some rate "*k_0_*". Cdc20 can either bind APC to form APC^Cdc20^ with some rate "*k_1_*" or get sequestered by the checkpoint through an emitted complex "*M*" (active protein(s)/complex(es) formed by Mad2, Mad3 and Bub3) with a rate *k_seq_* and form the sequestered Cdc20 complex "*MCdc20*". This complex can in turn also bind the APC with the same rate as the normal Cdc20 "*k_1_*". Bound APC^Cdc20^ and APC^MCdc20^ are both degraded but not at the same rate. The active APC^Cdc20^ is degraded at some rate "*k_2_*" whereas the checkpoint induced degradation is larger "*k_2_(k*_deg_*+1)*. A small APC independent Cdc20 degradation "*k_-0_*" and a small desequestering rate "*k_-seq_*" was also added for generality. Note that the total amount of APC and *M* are fixed to "*m_tot_*" and "*A_tot_*". We thus have the following set of equations:

$\begin{matrix} \frac{d\left[Cdc20\right]}{dt}=k_{0}+k_{-seq}\left[MCdc20\right]-\left[Cdc20\right]\left(k_{-0}+k_{seq}\left[M\right]+k_{1}\left[APC\right]\right) \\ \frac{d\left[MCdc20\right]}{dt} =k_{seq}\left[M\right]\left[Cdc20\right]-\left[MCdc20\right]\left(k_{-seq}+k_{1}\left[APC\right]\right) \\ \frac{d\left[APC^{Cdc20}\right]}{dt} =k_{1}\left[APC\right]\left[Cdc20\right]-k_{2}\left[APC^{Cdc20}\right] \\ \frac{d\left[APC^{MCdc20}\right]}{dt} =k_{1}\left[APC\right]\left[MCdc20\right]-k_{2}\left(1+k_{\deg}\right)\left[APC^{MCdc20}\right] \\ \frac{d\left[APC\right]}{dt} =-\left[APC\right]k_{1}\left(\left[Cdc20\right]+\left[MCdc20\right]\right)+k_{2}\left[APC^{Cdc20}\right]+k_{2}\left(1+k_{\deg}\right)\left[APC^{MCdc20}\right] \\ \frac{d\left[M\right]}{dt} =-k_{seq}\left[MCdc20\right]+k_{-seq}\left[MCdc20\right]+k_{2}\left(1+k_{\deg}\right)\left[APC^{MCdc20}\right] \\ \left[m_{tot}\right]=\left[MCdc20\right]+\left[M\right]+\left[APC^{MCdc20}\right] \\ \left[apc_{tot}\right] =\left[APC\right]+\left[APC^{Cdc20}\right]+\left[APC^{MCdc20}\right] \end{matrix}$(2a-h)

**2.1 Motivation for the use of ODEs**

Given the nature of the overall inhibiting mechanism, by emitted complexes [1], we previously showed [2] that using a system of ODEs is sufficient for modeling the budding yeast checkpoint.

**2.2 Some model clarifications**

*APC attachment rate (k_1_)*: The reason for using the same rate APC attachment rate for the inhibited and uninhibited Cdc20 is that there is no evidence whatsoever of impaired/improved binding to the APC of the inhibited complex. One could argue that the APC binding domain on Mad3 might contribute to a speedier APC binding of the MCC compared with Cdc20, This seems less likely however since Mad3 needs the APC to bind to Cdc20 [3].

*SAC enhanced degradation (k_deg_)*: There are two ways to explain the SAC induced increased degradation rates of Cdc20 in the active checkpoint [4, 5]. Either the checkpoint increase the rate explicitly [5, 6] or the increased rate is just a byproduct stemming from the fact that the APC degrades Cdc20 in M-phase (Cdc20 transcription is up-regulated in M-phase [3]). Here we assume a combination of the two. Thus the SAC increase the degradation of Cdc20 but not in a directly proportional way to the observed increase (~4 → ~10 times increased degradation [4, 5]). To confirm the independence of the solution from the degree of direct SAC degradation we fixed the inhibition rate and varied the ratio of direct versus indirect degradation for different levels of direct degradation (see figure S2). We see that the trend stay in all cases except the most extreme ones.


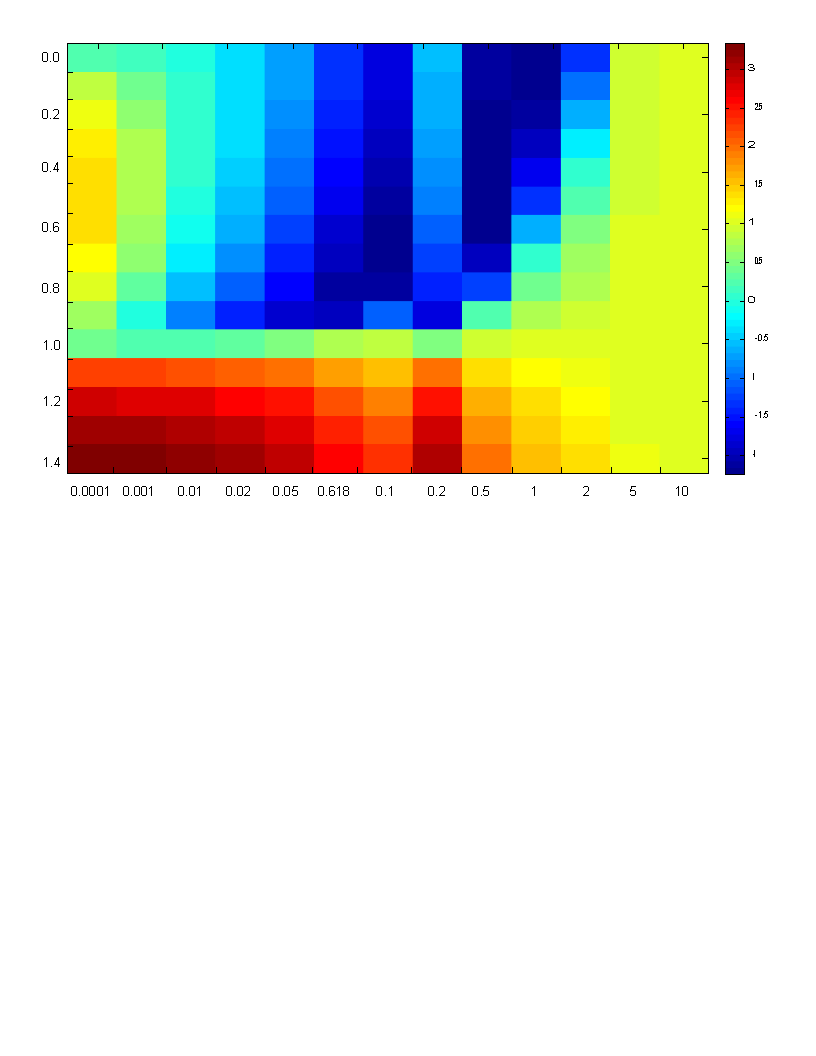


**Figure S2: Varying the fixed vs. direct degradation**

X-axis is the direct: indirect degradation ratio. Y-axis is the fraction of the direct degradation which is "on". The trend where there is an intermediate minimum around 1 is visible in all cases except the most extreme "direct" ones (to the right).

**2.3 Numbers and rates used in the model:**

*A_tot_*: The total number of APC complexes was set to 100. The reason for this is that the APC components with the lowest copy-numbers, Cdc23 and Cdc26, are ">50" and ~80. The numbers are from the "proteome" database[[1]](#footnote-2).

*M_tot_*: "*M_tot_*" corresponds somewhat roughly to the mean number of Mad2, Mad3 and Bub3 and is set to 3000 based on approximate values from the proteome database (as above) and from the estimations by Poddar et al [4]. Note that the exact value isn't that important since the effective sequestering rate is formed by the product between the amount of available *M* and the sequestering rate *k_seq._* which is varied during our screen (see below). *k_0_* and *k_-0_*: The steady-state number of M-phase Cdc20 is estimated to ~2200 [4] and the half time is estimated to ~7.5 min [5]. This means that the influx should be ~3.4 [molecules/s]. For simplicity we set the value to 10 [molecules/s]. There is no evidence that Cdc20 is degraded in any APC independent manner yet it seems plausible that it is possible that some (small) APC-independent Cdc20 degradation exists. Fore generality we therefore included an APC independent Cdc20 rate which was set to be 25 times lower than the APC dependent one.

*k_seq_* & *k_-seq_*: *k_seq_* is the effective inhibition rate which is controlled by the SAC. Hence it was varied over 6 orders of magnitude in our initial screen. We here assume that SAC sequestering is efficient i.e. *k_seq_>>k_-seq_*. We therefore assign *k_-seq_*, the desequestering rate, a comparatively small value.

*k_2_* and *k_deg_*: These rates control the ratio between indirect and direct checkpoint mediated degradation as explained above.
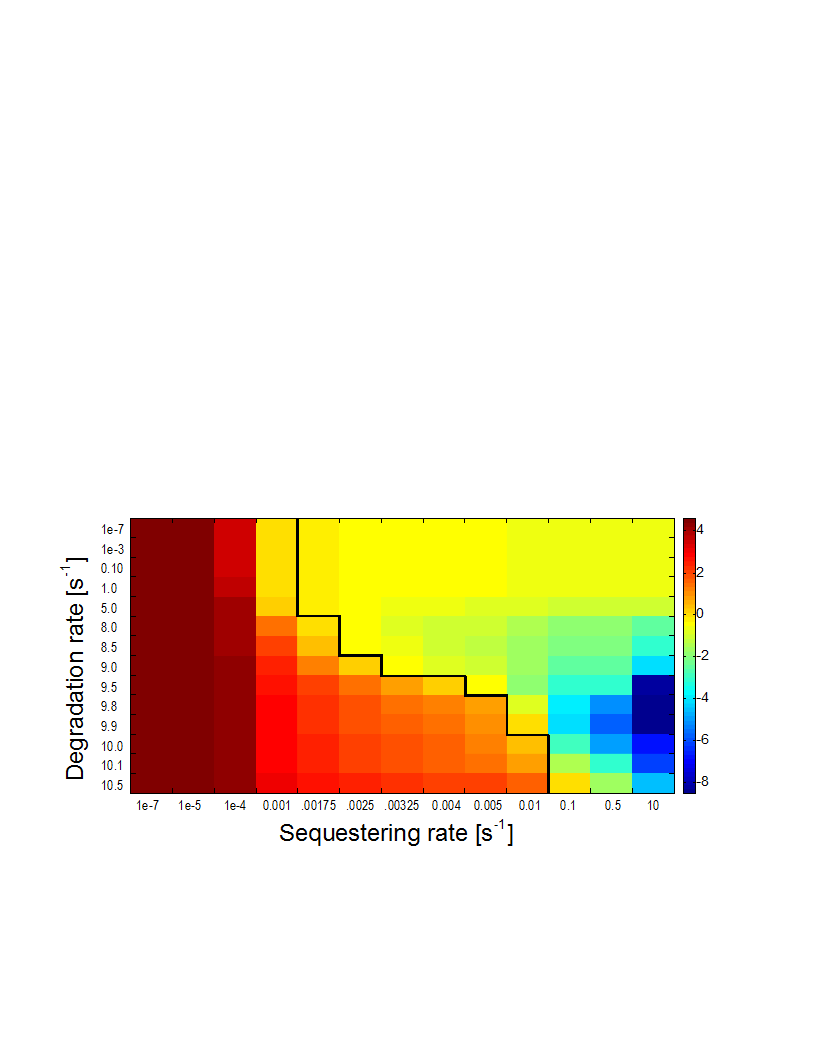


**Figure S3: Steady state level of log[APC^Cdc20^], when the SAC is on as a function of varying sequestering and degradation rates**

Note that the log([APC^Cdc20^]), values to the right of the black line represent an inhibition of >99%. The sequestering rate unit is [molecules^-1^s^-1^] and the degradation rates are X-times larger than the “natural” Cdc20 degradation *k_2_* (i.e. “1” means twice as large degradation “2” means three times as large etc…) the unit is also [molecules/s^-1^]. Colorbar represents log([APC^Cdc20^])

**2.4 Inhibition, reactivation times and noise resistance**

Previously, we used the total Cdc20 inhibition and the Cdc20 reactivation time (after the final kinetochore connected to the chromosomes) [1] as well as resistance to noise in the Cdc20 production [2] to determine whether certain classes of SAC mechanisms were plausible. In order to determine whether the current combination of sequestering and degradation was within these previously defined physical constraints of the SAC we calculated the level of APC^Cdc20^, the reactivation times and the noise resistance for all different sequestering and degradation rates. We found that for all cases were the APC^Cdc20^ inhibition is >99% (figure S3), both the reactivation times (figure S4) and the noise resistance (figure S5) was satisfactory. When it comes to determine what constitutes a satisfactory noise resistance we calculated the systems tolerance to a noise pulse in the Cdc20-production (as previously described [2]) and compared it with the noise tolerance of a “pure” degradation system. In all cases the added sequestering improved the noise tolerance significantly. Note that the optimal sequestering and degradation rates found (“8X” and ~0.00325 molecules^-1^s^-1^ see section 7.1) give a noise tolerance >100 times that of a pure degradation system (or >200s noise pulse tolerated).


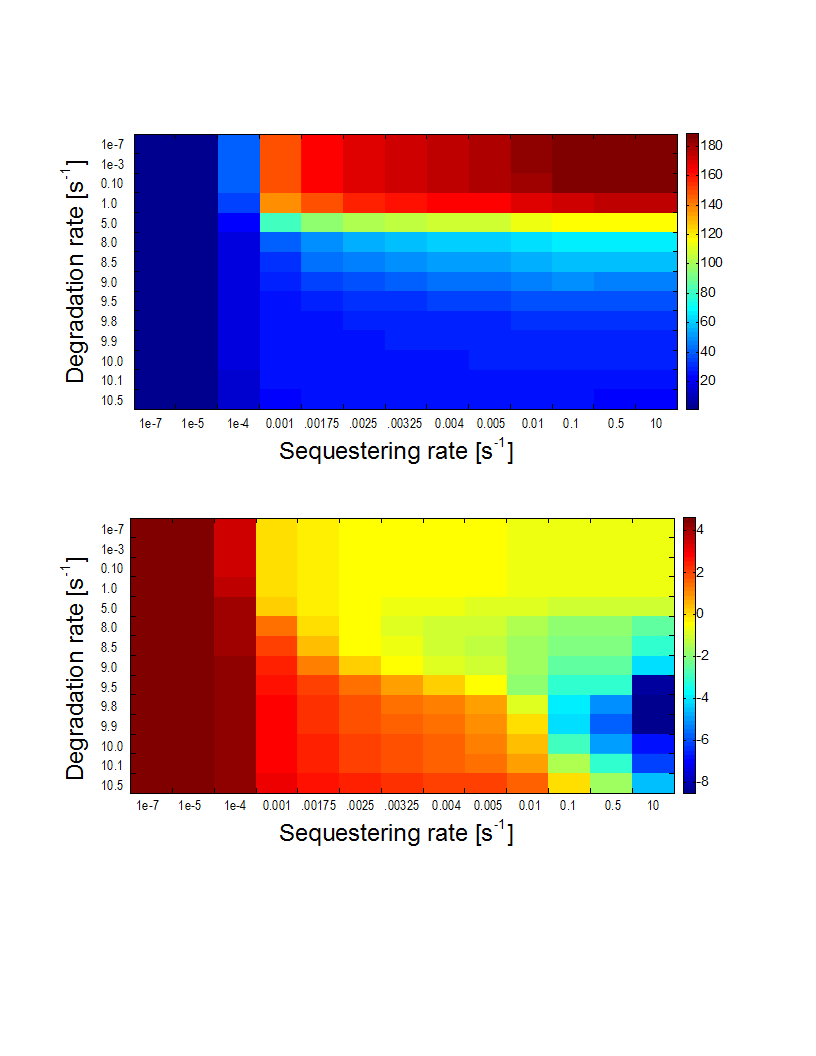


**Figure S4: Reactivation times**

After a steady state was reached the sequestering rate was turned off and the time to reach 90% of the maximal APC^Cdc20^ activity was measured. The reactivation time for the left most values (here 0s) was not possible to calculate since their steady state values were already over 90% of the total maximal APC^Cdc20^ activity due to poor sequestering. All other values were lower than 200s. Colorbar units are in seconds all other units as in figure S3.


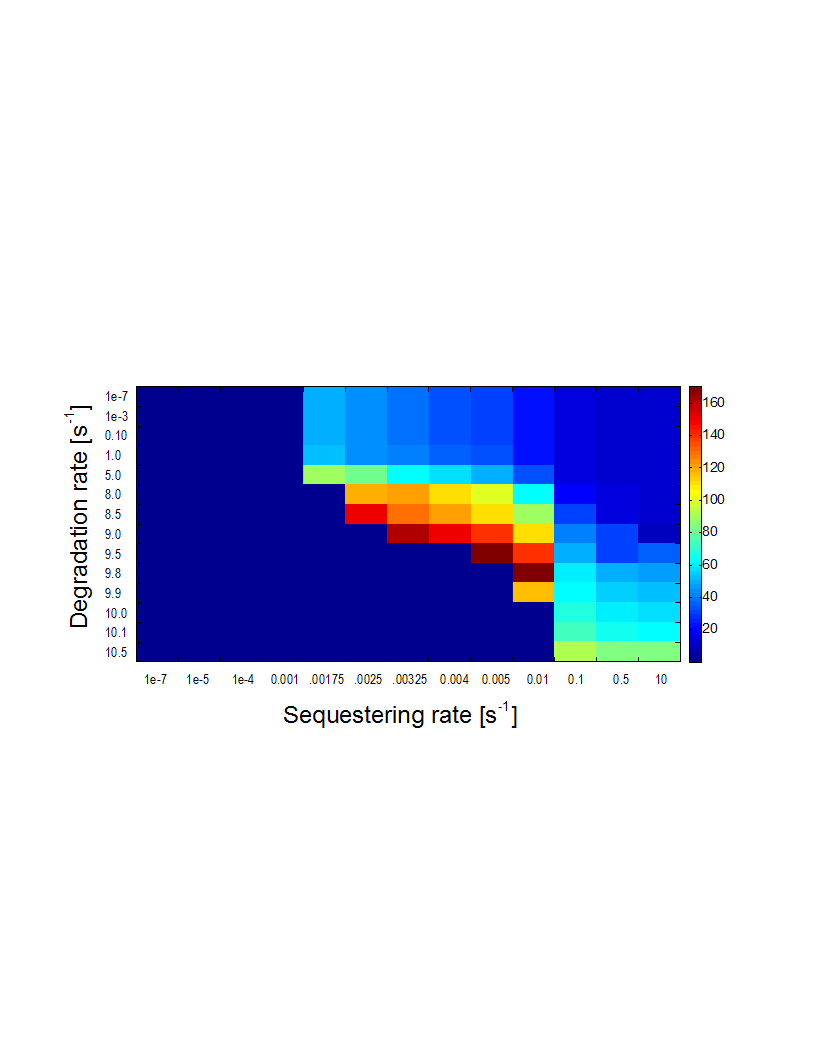


**Figure S5: Noise resistance**

After a steady state was reached we simulated a noise pulse in the Cdc20-production and measured the response of the system using our previously defined noise measure “ξ” (see [2] for details) for all cases where the APC^Cdc20^ inhibition is >99% (compare w. supp. fig. 3). We here show how many times longer noise pulse the system can handle compared with a pure degradation system given a reactivation time of 200s and 100 times serquestering (a degradation system with these constraints can handle a 2s pulse, see [2] for details). The colorbar units are [X-times better noise buffering than a pure degradation system], all other units as in figure S3.

**3 Correlation between the APC^Cdc20^ concentration and the chromosomal segregation error**

A simple flow chart of anaphase activation and chromosome separation looks like this:

(3)

We want to understand how the level of active APC^Cdc20^ affects the rate of chromosomal separation. A first assumption is the relationship between the APC^Cdc20^ and the chromosome separation rate is monotonic, that is: An increase in APC^Cdc20^ corresponds to an increase in the chromosomal segregation rate. This holds true since we know that higher APC^Cdc20^ leads to higher chromosomal error rates and there's no known mechanism that can even temporarily reverse that. Also conceptually it doesn't make any sense to have an inverse reaction.

In order to understand the nature of this monotonous relationship the process behind chromosome separation needs to be examined to some more detail. Unsurprisingly, the “real” mechanism behind the chromosome separation is more complicated than indicated above (Supp. Eq. 3). Numerous feedback loops exists between Mps1, APC, Cdc20, Esp1, Pds1, Scc1, Clb2, Cdk1 and Cdc14 which might regulate the proportionality between APC^Cdc20^ activity and Scc1 degradation. Apart from degrading Pds1 APC^Cd20^ also degrade Mps1 [6], a key upstream activator of the SAC which creates a feedback loop that fastens the deactivation of the SAC and promotes the metaphase to anaphase transition. Cdk1 on the other hand phosphorylate and stabilize Mps1 [7]. Cdk1 activity is downregulated once Cdh1 degrades Clb2 [8]. Pds1 inhibits Cdh1 activity [9], and thus prevents premature Clb2 degradation. Esp1 on the other hand promotes Cdh1 activity through Cdc14 [10]. Moreover Cdk1 activity promotes both the tethering of Esp1 to Pds1 and the nuclear localization of the Pds1-Esp1 complex [11] as well as Pds1 inhibition [12] (see figure S6).


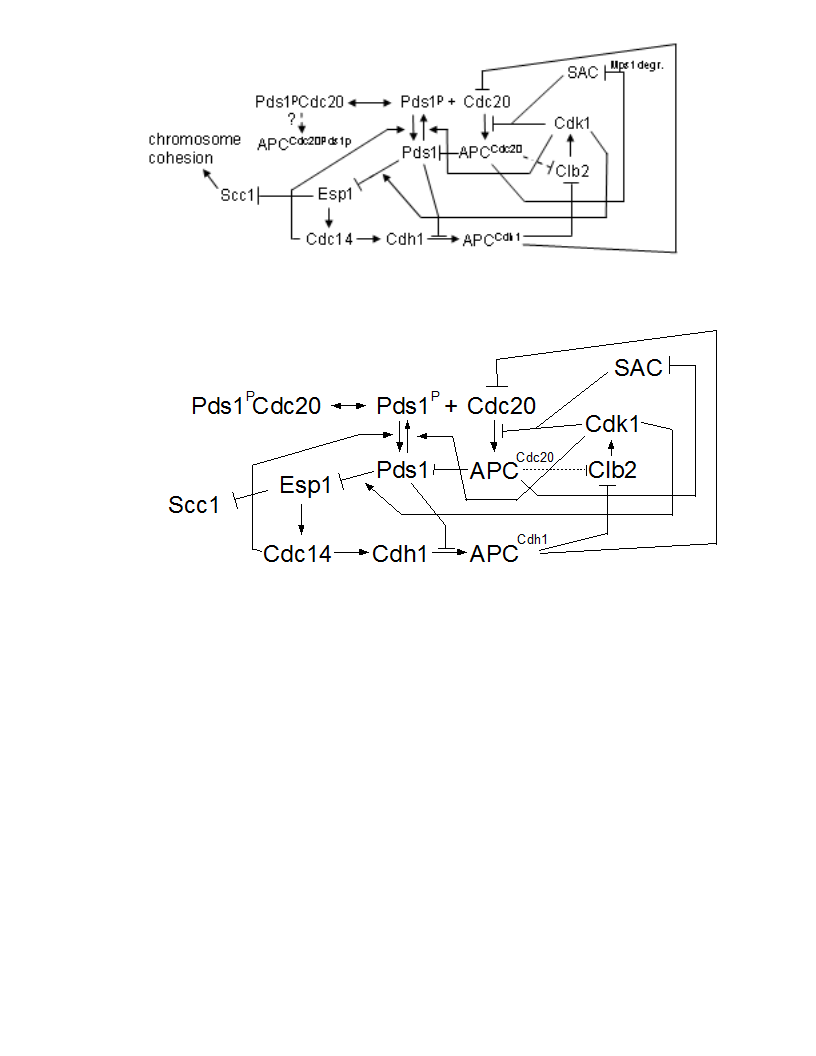


**Figure S6: The reactivation network**

Part of the reactivation network, see text for more details. Note that this picture is by no means complete, additional feedback loops exist in this network. Sic1, for instance, also interact with both Clb2 and Cdc14 [13].

Void of a thorough analysis of this system, which is beyond the scope of this paper, the monotonous relation between the chromosome segregation rate and the APC^Cdc20^ can be generalized into three different regimes, representing a missegregation rate which increases linearly or faster/slower with the APC^Cdc20^ (see figure S7):

*k<1*: If we first assume that the missegregation rate is hyper-sensitive to changes in APC^Cdc20^ (figure S7: k<1, red line). This scenario seems to be supported by some of the biological data where at least two feedback loops seem to speed up the separation of the chromosomes once a small fraction of APC is active (APC^Cdc20^→Mps1 degr. →SAC deactivation→ APC^Cdc20^ and Esp1→Cdc14→Clb2 degr. →Esp1, see also figure S6). On the other hand this alternative would make the checkpoint extremely unstable and it would imply that any mistake in the stalling would make a missegregation event more likely. If we compare this case with the detachment mechanism where any mistake does cause a missegregation event we see that the relative severity of the stalling is much less pronounced than that of the detachment. This suggests that the system does not operate in this regime.

*k>1*: In this alternative the missegregation rate is robust to changes in the APC^Cdc20^ (figure S7: k>1, green line). Conceptually this is an appealing thought since it would provide an additional failsafe mechanism for inhibiting the APC. There are however no known direct biological mechanisms supporting such a mechanism unlike the case when k<1. A k>1 would also effectively create a “back-up” checkpoint independent of the SAC, something that seems less likely given that we don’t have any evidence of such a mechanism. Moreover it would possibly slow down the reactivation of the checkpoint.

*k=1*: Here we will use the linear relationship (blue line figure S7). The reason for this is twofold: It is the simplest possible choice and there is no apparent better choice to the best of our knowledge. We would like to emphasize that this generalization only serves as a rough approximation for the purposes of this paper (where it seems to work), it does not necessarily reflect the real reactivation system where it is both possible and plausible that $k\neq1$.

**Figure S7: The possible relations between the APC^Cdc20^ and the chromosomal missegregation rate**

The relation between the APC^Cdc20^ and the missegregation rate can be generalized as a function *y=x^k^*. If *k<1*, the relative increase of the APC^Cdc20^ needed for chromosome missegregation is larger than when *k>1*.

**4 The chromosome missegregation rates depends on the strain and assay**

We compared our results of the chromosome missegregation rates with previously reported ones (supporting table 1). Note that the ratio of two normal distributions does not have a normal distribution but rather a Cauchy distribution. In order to properly calculate the standard deviations of our experiments one needs to take this into account (please see section 6 for an explanation of how it is done). None if the papers we compared our results with had done this which makes a comparison somewhat difficult.

| **deletion** | **X-fold increase in missegregation rate** | **Standard deviation** | **Warren et al [14]** | **Indjeian et al [15]** | **Fernius et al [16]** | **ratio** |
| --- | --- | --- | --- | --- | --- | --- |
| ***bub1*** | 34.7 | 5.3 | 50.6 | (13.7) | 41 | 1.5 |
| ***bub3*** | 21.6 | 3.0 | 33.9 | (13.3) | - | 1.6 |
| ***mad1*** | 2.6 | 0.29 | 4.5 | - | - | 1.7 |
| ***mad2*** | 2.7 | 0.27 | 3.5 | 3.3 | - | 1.3 |
| ***mad3*** | 1.4 | 0.15 | 2.7 | - | - | 1.9 |
| ***sgo1*** | 24.1 | 3.7 | - | (5.7,4.7) | 32 | 1.3 vs. 1.4 |

**Supporting table 1**: A comparison of our CMR with previously measured rates. Our results are in column two and three. All the “previous results” without standard deviations represent a “deletion” chromosomal loss rate divided by a “wild-type” chromosomal loss rate. See text for more details about the interpretation.

The strains used in all the three other papers are derivatives of w303 used with a sectoring assay [17] whereas we used the ALF-assay and by4742 strain derivatives. There are two possibilities for the different rates observed. Either it is due to an experimental artifact or it is a strain/assay dependent difference. If it is an experimental artifact we do not expect to see the ratio between our results and the other to stay reasonably constant which it is (with the exception of Mad2, see below). We thus conclude that the different results most likely are due to a strain/assay effect.

The values from [14] do not include any standard deviations. We here calculated the missegregation rates by dividing the number of missegregated mutant colonies with the wild-type value. The number of colonies was however reasonably large (29-89) for all the strains except for Mad2 (only 9). A similar *Δmad2* rate was reported by Indjeian et al [15] but also this one was very noisy. This might explain the slightly different ratio for the *Δmad2* deletion. In this context it is worth noting that since the only known function of Mad1 is to activate Mad2 (it is sufficient and necessary for this activation [18]), it makes sense that the missegregation rate of *Δmad1* is equal to the rate of *Δmad2* as our results indicate. The values from [15] do include standard deviations. Unfortunately they are way too large for us to use the simplified version of the ratio distribution to calculate the standard deviation of the ratio, (see below). In order to calculate the ratio distribution we demanded that the distributions are strictly positive at a point six standard deviations from the mean. Here the *Δbub1* rate, for instance, is $41\pm22.1$ and the wild type rate is$3\pm1.5$. The only somewhat reasonable value was *Δmad2* which was$10\pm0.7$. Yet even this value is too noisy due to the wild-type variation. The values in the table are the reported number of mutant colonies divided with the wild-type number.

The values from [16] are the number of losses (colored colonies) per thousand counted colonies instead of the quotient. The reason for this is that they report an extremely low wild type count: only 7 reported wild-type loss event from a total of 13588 colonies. This means that any quotient will be extremely shaky. Both the amounts of *Δbub1* and *Δsgo1* colonies are more reasonable and their quotient is similar to our (lower right field in the table).

Worth noting in the context of the ALF-assay is the fact that there are many cases where deletion strains attract suppression mutations or even suppressor chromosomes [19]. The probability of this increases the more severe phenotype the deletion has. Practically this implies that when comparing experiments the ones which produce high and consistent rates should be favored in front of those with lower noisier rates. It also means that any result, at least theoretically, could be higher than its experimental value.

**5 Separation of the error sources**

When we use the measured errors we assume that the stalling and detachment-parts of the checkpoint are mutually independent in the sense that the direct activity of one doesn't interfere with the other. Even though some proteins (most notable Bub1) are involved in both mechanisms there no evidence that increased APC^cdc20^ inhibition affects the detachment rate of the microtubule and vice versa. Practically this means that we can calculate the "stalling" missegregation rates for Bub1 (and Bub3 [20]) if we know to what fraction of the measured Bub1 rate comes from the detachment part of the checkpoint. It was shown that Bub1 promotes detachment of faulty kinetochore-microtubule connections by activating Sgo1 through phosphorylation [16]. Moreover it was also shown that the Bub1 activation of Sgo1 is not complete in the sense that a bub1deltaK mutant (Bub1 mutant without its phosphorylation activity) is not as severe as the *Δsgo1* [16]. In fact, the ratio between the Bub1-activated part of the missegregation rate and the total Sgo1 rate was found to be approximately 25/32 [16]. This means that we can use this relationship and the *Δsgo1* and *Δbub1* missegregation rates to calculate how much of the *Δbub1* rate that goes to the detachment.

Initially the following values were obtained for the single deletions:

| Name | Rate | Standard deviation |
| --- | --- | --- |
| Sgo1_1 | 24.2 | 3.6 |
| Sgo1_2 | 23.9 | 3.7 |
| Bub1 | 34.7 | 5.3 |
| Bub3 | 21.6 | 3.0 |

Here “Sgo1_1” and Sgo1_2 represent two independent experiments. Now it was possible to calculate how much of our *Δsgo1* missegregation rate that comes from *Δbub1*:

$\left(\frac{sgo1\_1+sgo1\_2}{2}\right)\frac{25}{32}\approx18.9$ (4)

We can then divide the *Δbub1*-rate with this number and we have the stalling (non- *Δsgo1*) part of the *Δbub1* missegregation rate.

The *Δbub3* case is more complicated. Since the relation of the Bub3-detachment interaction has never been quantified we here assume that the Bub3 contribution to the microtubule detachment rate is proportionally the same as the Bub1 contribution. According to Fernuis et al, [16] approximately 25/41 of the total *Δbub1* rate is involved in the Sgo1 activation. In order to compare the *Δbub3* rate with these values we first rescale *Δbub3* according to the different strain/assay used in the previously mentioned paper [16]. Then it is possible to calculate how big fraction of Bub3 is involved in the Sgo1 activation we get the following:

$Sgo1\left[\frac{\left(\frac{25}{41}\right)\Delta bub3*1.6}{32}\right]\approx15.6$ (5)

Hence the following rescaled rates was obtained:

$$\begin{matrix} \Delta bub1=1.8 \\ \Delta bub3=1.4 \end{matrix}$$

**6 About the ratio between two normally distributed functions**

Note this entire section is adapted from <http://www.mathpages.com/HOME/kmath042/kmath042.htm>

Let ***X*** and ***Y*** be two independent random variables and let *g(x)* and *h(y)* be their density functions. We also assume that *g(t)=h(t)=0* for all *t £ 0*.

Taking a random sample from ***X*** and ***Y***, the combined sample will represent a point *(x,y)* in the *(****X****,****Y****)* plane. The joint density function of this point *(x,y)* is the product of the two univariate densities *g(x)h(y)*. In the same way the probability that a random joint sample would fall within some particular region is just the integral of *g(x)h(y)* over that region.

**Figure S8: *q* and *q+dq* in the (*X*,*Y*)-plane**

We can now denote "*q*" such that the set of all points for which the ration *y/x* equals a constant *q*. This *q* makes up a line in the *(****X****,****Y****)* space. The probability that a random sample have a smaller ratio than *q* is then just the integral of *g(x)h(y)* over the regions i.e. under the "*q*"-line:

$$P\left(\frac{y}{x}\leq q\right)=\int_{x=0}^{\infty} \int_{y=0}^{qx} g\left(x\right)h\left(y\right)dydx \left(6\right)$$

In the same way the incremental probability that the ratio is between *q* and *q+dq*, for some small *dq* can also be calculated (see also figure S8):

$$P\left(q\leq\frac{y}{x}\leq q+dq\right)=\int_{x=0}^{\infty} \int_{y=qx}^{\left(q+dq\right)x} g\left(x\right)h\left(y\right)dydx \left(7\right)$$

Since *g(x)* and *h(y)* are independent *g(x)*, *g(x)* can be moved from the inner integral. If we also assume that the increment *dq* is small enough then *h(y)* will be almost constant and *y~qx* this gives us:

$$dF\left(q\right)=\int_{x=0}^{\infty} g\left(x\right)h\left(qx\right)\left[\int_{y=qx}^{\left(q+dq\right)x} dy\right]dx \left(8\right)$$

We now denote this quantity "*dF(q)*" since it is the incremental change of the cumulative function *F* for an incremental change *dq* in the ratio. Evaluating this integral and dividing both sides with *dq* gives us:

$$f\left(q\right)=\frac{dF\left(q\right)}{dq}=\int_{x=0}^{\infty} xg\left(x\right)h\left(qx\right)dx \left(9\right)$$

By taking the limit when *dq→ 0* we this get the density function *f(q)* for the ratio ***Y****/****X***.

If ***X*** and ***Y*** are normal distributions with ***X****>0* at zero, then *f(q)* will be a Cauchy distribution. On the other hand if *g(x)* vanishes at zero then we can simplify this expression further and calculate the mean and standard deviation for the ratio distribution. In our case this is a reasonable assumption since we will exclude any plate with zero colonies and no plate will obviously have a negative amount of colonies. Practically we will exclude any experimental value which has a non-zero value six standard deviations from the mean.

From Eq. (6) we get the following expectation value:

$$E\left(q\right)=\int_{q=0}^{\infty} q\int_{x=0}^{\infty} xg\left(x\right)h\left(qx\right)dx dq \left(10\right)$$

which is the same as

$$E\left(q\right)=\int_{x=0}^{\infty} \left(\int_{q=0}^{\infty} qxg\left(x\right)h\left(qx\right)dq\right)dx \left(11\right)$$

here x is constant in the inner integral and since *y=qx* we have that *dy=xdq* and *q* and *y* ranges from *0* to infinity. We can thus replace *q* with *y/x* and *xdq* with *dy* and let the inner integral variable be *q*.

$$E\left(\frac{y}{x}\right)=\int_{x=0}^{\infty} \left(\int_{y=0}^{\infty} \frac{y}{x}g\left(x\right)h\left(y\right)dy\right)dx \left(12\right)$$

This expression can now be separated into an *x*-part and a *y*-part and since *x* and *y* are independent we can write the double integral as their product.

$$E\left(\frac{y}{x}\right)=\left(\int_{y=0}^{\infty} yh\left(y\right)dy\right)\left(\int_{x=0}^{\infty} \frac{1}{x}g\left(x\right)dx\right)=E\left(y\right)E\left(\frac{1}{x}\right) \left(13\right)$$

Following the same reasoning as in equations five to eight we can also calculate *E(q^2^)*, the expectation value of *q^2^* which gives us the standard deviation of the ratio.

In our case we have one wild type strain, which is normally distributed and corresponds to *g(x)* and one deletion mutant, which is also normally distributed and corresponds to *h(y)*. We can thus first calculate the means and standard deviations of *g* and *h* and then use formula eight and its extension to get the mean and standard deviation of the ratio. Practically we integrated our *g* and *h* numerically over a range of 12 standard deviations centered at the mean.

**7 The screen**

In the screen we have the following variables: the sequestration rates (6), the degradation rates (5), the external activations (5) and the variable weights (depends on the amount of edges in the kinetochore network).

We used the following sequestration complexes: Bub3-Mad2-Mad3 (MCC), Mad2-Mad3 (putative complex), Bub3-Mad2 (speculative complex), Bub3-Mad3 (complex with unknown function), Mad2 (known sequestering complex) and Mad3 (putative complex). Out of the possible combinatorial possibilities we only excluded Bub3 since no known binding between Cdc20 and Bub3 exists.

For the degradation, the following complexes were assumed: Bub3-Mad2-Mad3 (MCC), Mad2-Mad3 (putative complex), Bub3-Mad2 (speculative complex), Bub3-Mad3 (complex with unknown function), and Mad3 (putative complex). Out of the possible combinatorial possibilities we here excluded Bub3 and Mad2 since they cannot promote the Cdc20 degradation alone.

We also varied the external activations for all the proteins simulating either their endogenous ability to reach the kinetochores or some external activation (Mps1 or Ipl1).

Each kinetochore network has between 1 and 10 edges (see figure S1). When varying the edges the exact number of iterations will therefore depend on the complexity of the network. If, for instance we vary the kinetochore weights between 3 values then a network with X edges will have 3^X^ combinations.

**7.1 Finding the optimal APC^Cdc20^ minimum**

Our first task was to determine which APC^Cdc20^-minima were optimal for the screen. We ran an initial screen were we varied the sequestering rate and adjusted the degradation rate so that we would get maximal APC^Cdc20^ inhibition (see figure S9 for the relation between the sequestering and degradation rates giving the optimal APC^Cdc20^ inhibition). Each such point was then screened varying the other parameters to determine the optimal one (data not showed). All screens gave the same result with a sequestering rate of (~5.4nM^-1^s^-1^, calculated from a nuclear volume of ~10^-15^m^3^ and an original rate of 0.00325molcules^-1^s^-1^) and a degradation rate of ~.092s^-1^ regardless of complexes/networks used. This is not surprising considering that the relative derivative in the "APC^Cdc20^-sequestering-degradation" plane is determined by the location of the minimum. This derivative also determines the relative differences in the rates needed to change the predicted missegregation rates. This in turn depends on the experimentally determined missegregation rates and on the increments used in the screen. The relative contribution of each complex will however remain the same. What might change are the constant component and the ratios of the contributing complexes to some degree. This means that our result might not be quantitative with respect to determining the "real" sequestering rate but rather tells us the structure of the network with some indications of their relative importance.

**Figure S9: Relation between the degradation and sequestering rates for the minimal APC^Cdc20^**

We see that the stronger sequestering the faster degradation is needed to keep the APC^Cdc20^ down. Units: X-axis molecules^-1^s^-1^, Y-axis 0.0038+0.0618 times the indicated number s^-1^.

**7.2 Choosing the screen weights/rates**

In the screen we had to choose values for all the kinetochore weights, the external activations as well as for the sequestering and degradation rates. Since all the final outcomes from any system were normalized to the wild-type missegregation rate we only need to look at the relative differences between the weights/rates. Ideally one wants to vary those over as large range as possible to cover all possible outcomes. Practically we found that varying the kinetochore weights between 0.1 and 1 and the sequestering and degradation rates between 0.01 and 1 (see below) was sufficient to get results which gave us results which fitted the known missegregation rates. Note that in the second step of the screen (below) when the rates were varied between 0 and 0.1 we used the following values [0 0.01 0.1].

The constant sequestering and degradation rates were set to 0.01. It is not known to what degree the “non-SAC” mechanisms contribute to the inhibition and by setting varying all other rates we effectively fitted this “outside” contribution to our solutions. The relative fraction to the total sequestering and degradation from these constants rates might vary with the chosen APC^Cdc20^ minimum and with the missergregation assay (see section 9).

**7.3 Screening the system**

Due to the amount of variables we decided to do the screen in two steps. First we fixed all the sequestering and degradation rates as well as the external activation of Mad1, Mad2 and Mad3. The kinetochore weights and the external activation of Bub1 and Bub3 were varied. The rates for the proteins/complexes that are known to contribute to the SAC, Bub3-Mad2-Mad3 (seq. and deg.), Mad2(seq.) and external activation of Mad1 and Mad3 was set to 0.1 whereas the putative rates (the rest) were set to 0.01. The reason behind this was that we wanted to catch any beneficial impact from the putative complexes while ensuring that the known complexes were present. To compensate for any detrimental effects from included but non-contributing complexes we set the threshold as high as 0.2, meaning that the calculated maximal difference between the experimental and estimated data could be as high as 20%. The constant sequestering and degradation rates were set to 0.01.In this first step we went through 12273124 possible networks including all the kinetochore networks. This gave us 313 solutions divided to 116 kinetochore networks.

In the second step we started with the solutions from the first one and varied the parameters further..The known rates were varied between the values 0.01 to 1 and the putative ones between 0 and 0.1. The reason that we varied the putative values to a lower relative level (0.1 vs. 1) is twofold: First, from a practical point of view a combination with one or two additional values increase the simulation time exponentially and make the entire simulation impracticable. Second, such an addition would not add anything to the solutions as the only set of solutions that we potentially miss is one where the putative weights completely overshadow the known inhibiting mechanisms. Needless to say, that is a very unlikely scenario. In the second step we kept the kinetochore weight from the previous solution for all networks, if more than one set of weights were found, we reused both (in no case did we found more than 2 weights). We also allowed either Bub1 or Bub3 as the most upstream component according to the previous solution. We did not however allow for simultaneous Bub1 and Bub3 activation since it is clear that one of them precede the other [21,22].

Solving for these values we went through another 17307648 possible networks with a threshold of 5%. We now found 105 solutions representing 24 different networks. In the screen itself we did not distinguish between solutions producing equivalent or near equivalent results as long as they are unique (see figure S10 for an explanation of the origins of such redundancies). After the screen however, we reduced all redundant solutions so that only the simplest possible significant combinations of sequestering and degrading complexes were considered (see figure S10C-D). This gave us a total of 82 solutions divided into 20 kinetochore networks (figure S11).


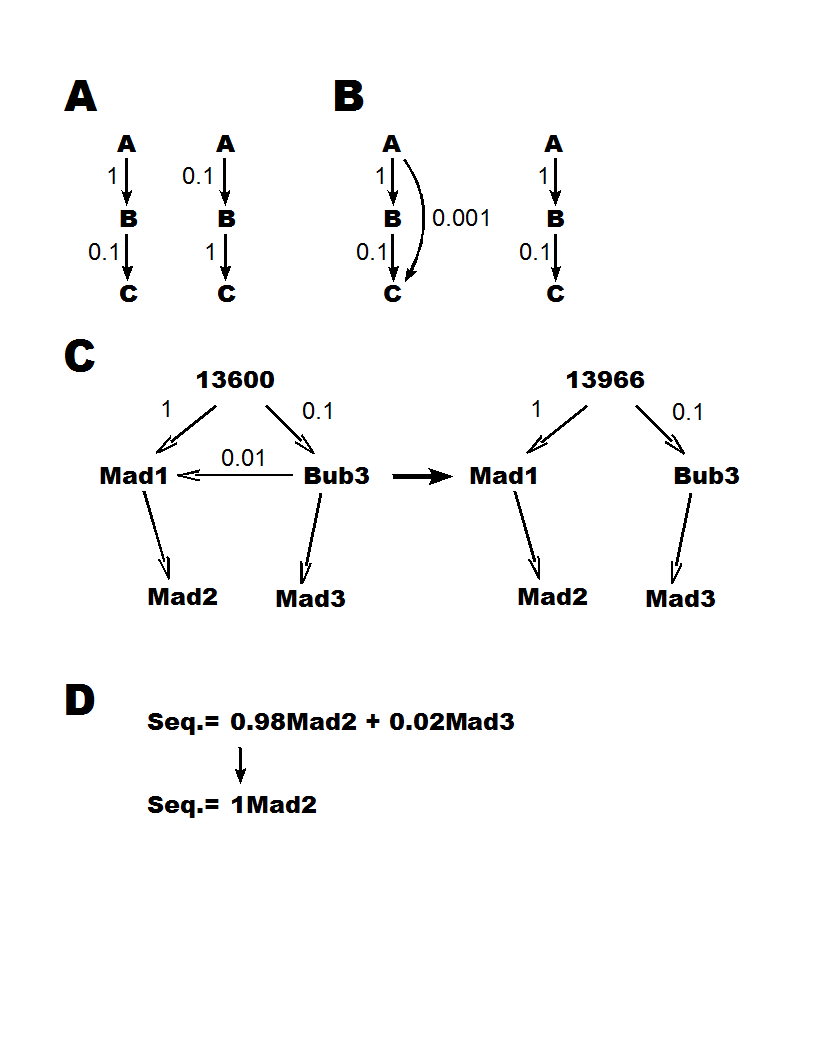


**Figure S10: Examples of redundant networks and edges**

**A**. In this hypothetical example the effect on the output variable “C” is the same from in both cases. **B**. Also in this hypothetical network the two networks are considered the same since the A → C edge is weak (1%) compared with the B → C edge. All edges contributing less the 5% of the “total” were considered redundant and were removed from the set of solutions. **C**. An example from the final set of solutions in the screen: Network 13600 contained an edge from Bub3 → Mad1 with a weight less than 1% of the total contribution to Mad1 (0.01/1.01). Due to the relative insignificance of this edge it can be removed and the solution is reclassified as 13966 (while keeping its unique set of sequestering and degradation complexes). **D**. Removal of redundant non-contributing complexes: If all other parameters, including the kinetochore network, external activations and the predicted double deletions, were identical between two solutions any non-significant sequestering and degradation complexes were removed. Note this was only done when the added complex did not in affect the predictions.


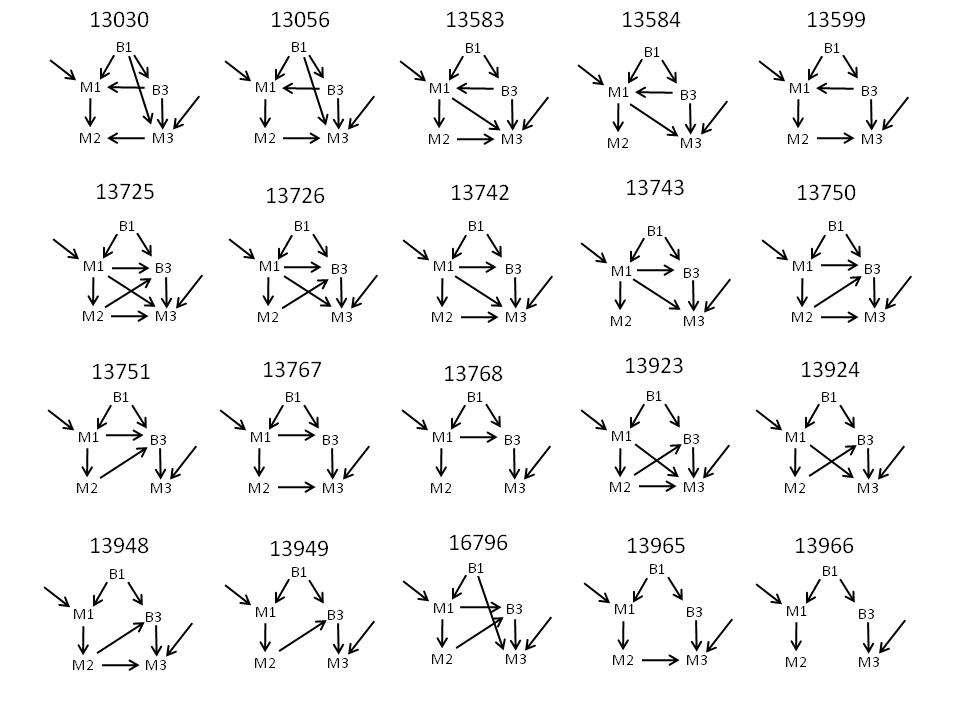


**Figure S11: 20 networks that predicts the single deletion missegregation rates**

The only network that correctly predicted the solutions without the Bub3-Mad2 complex is number 13966 (lower right). Network number 13965 (also lower right) belongs to the group of solutions that predict the correct solutions using the Mad2-Bub3 complex.

**8 Analysis of the uniqueness of our solution network**

Given our specific network solution, are there any other networks that might produce the same phenotype? Or in other words: Is our solution unique within the model framework or redundant?

Our solution can be generalized as a specific kinetochore network with a connected function for some of the kinetochore components (see figure S12).


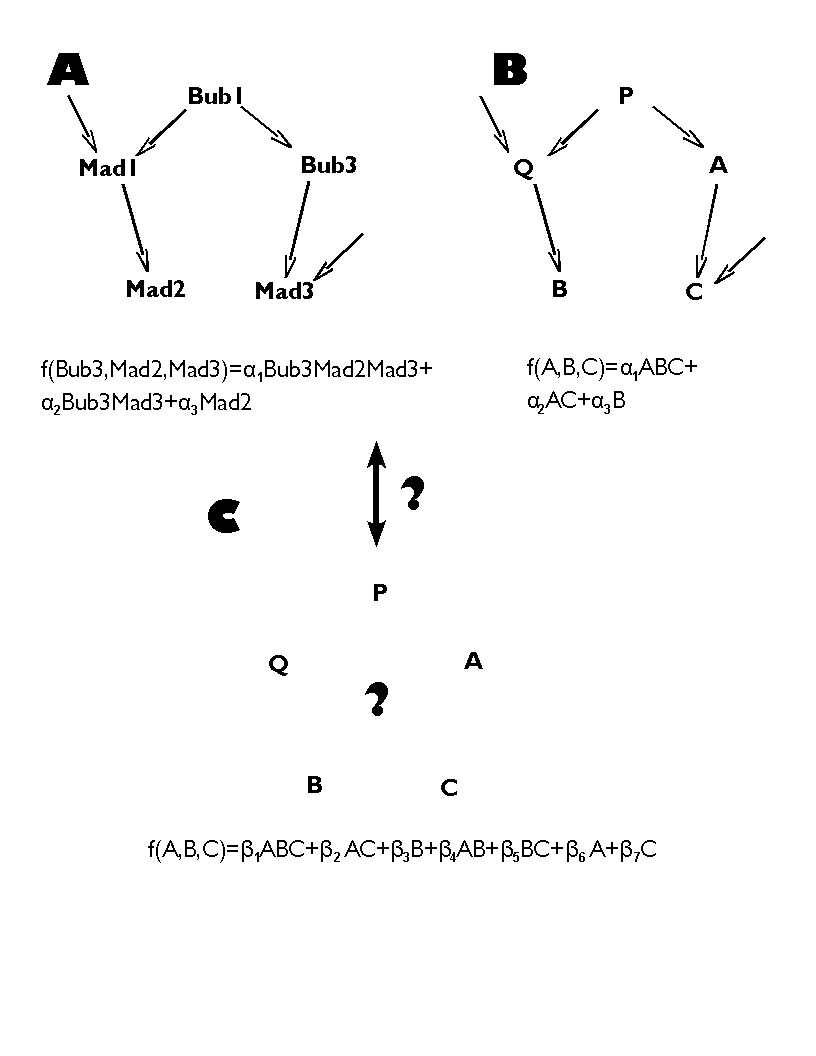


**Figure S12: A generalization of the resulting network**

A. Our network from the screen is somewhat generalized in the sense that we here represent the Cdc20 inhibition as one general function f instead of an interplay between two mechanisms (sequestering and degradation). B. We further simplify the notations. C. Is there another network for any set of kinetochore interactions and Cdc20 inhibiting complexes which is both different from our network and give the same phenotypes?

Note that we generalize the sequestering and degradation to one function. This assumption means that we start at the wild-type (optimal) level of APC^Cdc20^ and any decrease in sequestering and/or degradation will lower the overall inhibition in the same way. Moreover it ought to be simpler to mimic a function depending on only one variable so if we can show that no redundancies exist in this case it most likely holds also for the real case.

We here propose to solve this problem in two parts: First we show that regardless of the kinetochore network the given phenotypes have to include only the found inhibiting complexes. Second, given the same inhibiting complexes, we show that also the kinetochore networks have to be the same.

The inhibiting complexes are identical regardless of the kinetochore networks.

In order to show this we assumed that the known inhibiting complexes (*ABC, AC* & *B*) with their constants (*α_1_, α_2_* & *α_3_*) sums up to some rate causing a wild type level of inhibition (equation 12 LHS). The inhibiting complexes for some arbitrary network must always sum up to the same wild-type level (the same total inhibiting rate, equation 12 RHS). Note that if we can show that all the *β*s, denoting the putative complexes of the arbitrary network, except *β_1_, β_2_ and β_3_* are zero then the inhibiting complexes are the same.

$\alpha_{1}ABC+\alpha_{2}AC+\alpha_{3}B=\beta_{1}A'B'C'+\beta_{2}A'C'+\beta_{3}B'+\beta_{4}A'B'+\beta_{5}B'C'+\beta_{6}A'+\beta_{7}C'$ (14)

Note that we don't know the values of neither the unknown constants *β* nor the *A'*, *B'* and *C'*, only that their sum is normalized to the same wild-type value as our known network. We also assume that all involved complexes are significant, that is they contribute with at least 5% of the total signal. From our experimental analysis we have the following set of deletions:

$$\Delta A, \Delta B, \Delta C, \Delta BC$$

If we now simulate the deletions of *A*, *B* and *C* we need to get the same solutions from the known and unknown solutions:

$\begin{matrix} \Delta A: \alpha_{3}B=\beta_{3}B''+\beta_{5}B''C''+\beta_{7}C'' \\ \Delta B: \alpha_{2}AC=\beta_{2}A''C''+\beta_{6}A''+\beta_{7}C'' \\ \Delta C: \alpha_{3}B=\beta_{3}B''+\beta_{4}A''B''+\beta_{6}A'' \end{matrix}$ (15 a-c)

Here, the right hand side *A*, *B* and *C*s might be different from the previous ones since we do not know whether any *A*, *B* or *C*-deletion affect the other components in the unknown kinetochore network. Next we "delete" *B* and *C*:

$\Delta BC: 0=\beta_{6}A'' \Rightarrow\beta_{6}=0$ (16)

but we also have that

$\Delta AB=\Delta BC=0 \Rightarrow\beta_{7}=0$ (17)

Next we use the fact that: $\Delta AC=\Delta A=\Delta C$ this implies that: $\beta_{4}=\beta_{5}=0$. We thus get the following relationship:

$\alpha_{1}ABC+\alpha_{2}AC+\alpha_{3}B=\beta_{1}A'B'C'+\beta_{2}A'C'+\beta_{3}B''$ (18)

Which is exactly what we wanted to show: the unknown system has the same set of inhibiting complexes as the known one.

Next we look at the kinetochore network given the fact that the inhibiting complexes are the same. Here we need to use some of the quantitative results from the missegregation assay. We have that:

$\Delta Q=\Delta B$ (19)

Given the fact that the function of Mad1 (*Q*) and Mad2 (*B*) are very well known (Mad1 function is necessary and sufficient for Mad2 activation) we assume that the missegregation rate from the double mutant *Δmad1Δmad2* equals that of either *Δmad1* or *Δmad2* (this assumption is further strengthened by the fact that $mad1mad3\approx mad2mad3$) that is:

$\Delta QB=\Delta Q=\Delta B\Rightarrow Q\to B$ (20)

The first equality means that *Q* and *B* are in the same pathway and the fact that *B* is part of the inhibiting complexes whereas *Q* is not implies that *Q* has to activate *B* not the opposite. If the opposite was true then *Q* has to affect some other inhibiting component (*A* or *B*) in a way that causes a significant difference. This, in turn would have meant that$\Delta Q\neq\Delta B$*.*

Next, we have the following:

$\Delta PA=\Delta P>\Delta A$ (21)

this means that *P* has to affect either *A/C* or *B*. the following is possible:

1. *P* affects only *B* $\Rightarrow\Delta PA>\Delta P$
2. *P* affects only *A*/*C* $\Rightarrow\Delta P\leq\Delta A$
3. *P* affects *B*/*Q* and *A*/*C* to some degree

Since both (i) and (ii) are not true *P* has to affect both *B*/*Q* and *A*/*C*. Next we ask ourselves whether P can affect B directly. If this is the case then $\Delta Q\neq\Delta B$, so the answer is no. Moreover we have that $\Delta P\leq\Delta Q\Rightarrow$*Q* or *B* must have some external activation. This has to be *Q* since if it is *B* then$\Delta Q\neq\Delta B$. The resulting interactions can be seen in figure S13a.


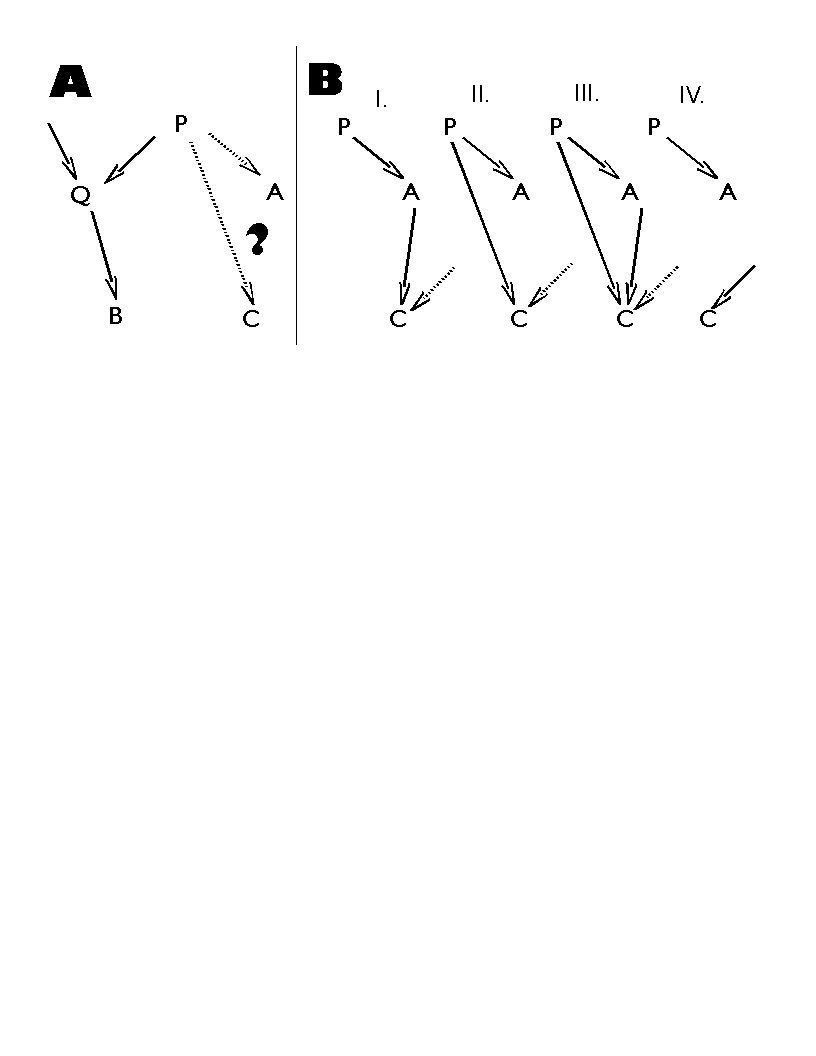


**Supporting figure13: Possible interaction between Bub1, Bub3 and Mad3**

A. We thus have the following interactions in the unknown network. Note that we only added what we know there might still be more interactions. B. Due to the nature of the inhibiting complexes in our solution (A and C always acting together) we cannot distinguish between any of the four possible solutions (I-IV). For biological reasons (see main paper) we are going to assume that "I." is the right one.

Next, we looked at the possible interactions which remained (see supporting table 2):

| Possible interaction | interpretation |
| --- | --- |
| *P → C* | Ok, see figure S13b |
| *Q → P,A,B,C* | Not possible would imply $\Delta Q\neq\Delta B$ |
| *B →* A | Not possible, see (i) below |
| *B → C* | Possible |
| *B → P* | Not possible, leads to a loop |
| *A → Q* | Not possible implies that $\Delta A\neq\Delta C$ |
| *A → B* | Not possible would imply $\Delta Q\neq\Delta B$ |
| *A → C* | Ok, see figure S13b |
| *C → A* | Not possible, see (i) below |
| *C → P* | Not possible, see (i) below. Might also induce a loop |
| *C → B* | Not possible would imply $\Delta Q\neq\Delta B$ |
| *C → Q* | Not possible, see (ii) below |

**Supporting table 2**: Possible interactions on the kinetochore

1. In the cases indicated here we use the fact that *P* and *A* (i.e. Bub1 and Bub3) are also involved in the detachment mechanism. This means that any proteins involved in activating them in a significant way would need to have a missegregation rate which is much larger than any non Bub-proteins have.
2. If there is an external C activation, as we have in the known network, then $\Delta A\neq\Delta C$

All in all, we get two possible networks (figure S14) we note that the 13965 was indeed in the screen and was there found not to fit the requirements when the more extensive relationship between sequestering and degradation was being used to discriminate the solutions. We thus conclude that no redundancies exist.


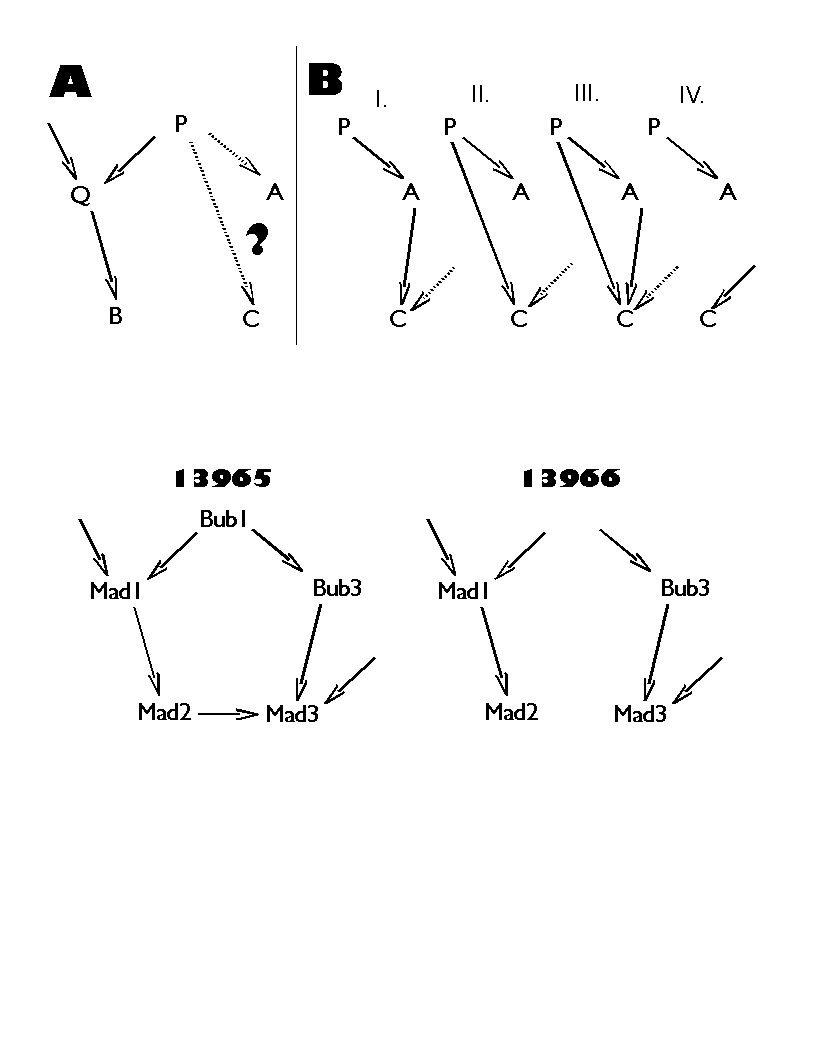


**Figure S14: The resulting kinetochore networks**

Note that the one we found is the simpler one that explains all the phenomena.

**9 The relative contributions from the inhibiting complexes**

In the one consistent topology (solution) most of the sequestering is done by Mad2, whereas the MCC and the Bub3-Mad3 is responsible for the degradation (see supporting table 3). The relatively small contribution of the MCC to the sequestering indicates that the MCC promoted degradation of the Cdc20 is rapid. The Bub3-Mad3 degradation is mostly due to the fact that Bub3-Mad3 degrades Cdc20 indirectly by binding Mad2-Cdc20 forming the MCC. Based on our results we cannot exclude the possibility of Bub3-Mad3 independent Cdc20 degradation. The fact that our results can be interpreted in two ways is a consequence of the fact that we do not account for specific reactions in our analysis but rather the relative contribution of the different complexes. Nonetheless, it is known that Mad2 is needed for efficient Cdc20 degradation [23,24] which means it is likely that Bub3-Mad3 promoted Cdc20 degradation comes from MCC formation. Note that is was recently suggested that Cdc20 might be degraded independently of Mad2 in human cells [25]. It is not known whether this result reflect the SAC in yeast since it seems to contradict the fact that Mad2 is needed for Cdc20 degradation [24]. It does however provide an alternative interpretation of our solution: The Bub3Mad3 contribution to the degradation would, if this model is correct, represent a Mad2 independent degradation of Cdc20 instead of the formation of the MCC (figure 7). Such a Mad2-independent degradation would entail a Mad2 dependent shuttling of Cdc20 to the Bub3-Mad3-APC (figure S14).

|  | SEQUESTERING (relative fractions) | | | | | | | |
| --- | --- | --- | --- | --- | --- | --- | --- | --- |
| Number | MCC | M2M3 | B3M2 | B3M3 | B3 | M2 | M3 | Const. |
| 13966 | 0.03 | 0 | 0 | 0 | 0 | 0.51 | 0 | 0.46 |
|  | DEGRADATION (relative fractions) | | | | | | | |
| 13966 | MCC | M2M3 | B3M2 | B3M3 | B3 | M2 | M3 | Const. |
|  | 0.01 | 0 | 0 | 0.50 | 0 | 0 | 0 | 0.49 |

**Supporting table 3:** The different contributions from the diffusible complexes to the total inhibition. Note that MCC is the same as Mad2-Mad3-Bub3. B1=Bub1, B3=Bub3, M1=Mad1, M2=Mad2 and M3=Mad3. networks in the same box represent the same kinetochore solution (i.e. 13426 and 13966)

Another noticeable thing is the relatively large contribution from the constant term. Given the general nature of our approach it is not possible to determine the exact source of this constant term. The most likely explanation is a contribution to the Cdc20 inhibition from other sources such as Cdc20 phosphorylation.

It is however possible that the degree of ‘constant’ contribution is dependent on our analysis itself. When we fixate the location of the APC^Cdc20^ minima used in the screen we rely on the specific measured missegregation rates for the single deletions (see above). This means that using a different strain/assay (with a higher overall missegregation rate) might change the relative contribution from the constant term. Preliminary screen using missegregation rates from Warren et al [14] (data not shown) shows that the contribution from the constant fraction does change to some degree while the nature of the consistent topology seems to stay the same. Due to the high noise level in these rates and to the fact that we cannot use any double deletions to determine one specific topology such results are however, hard to interpret.


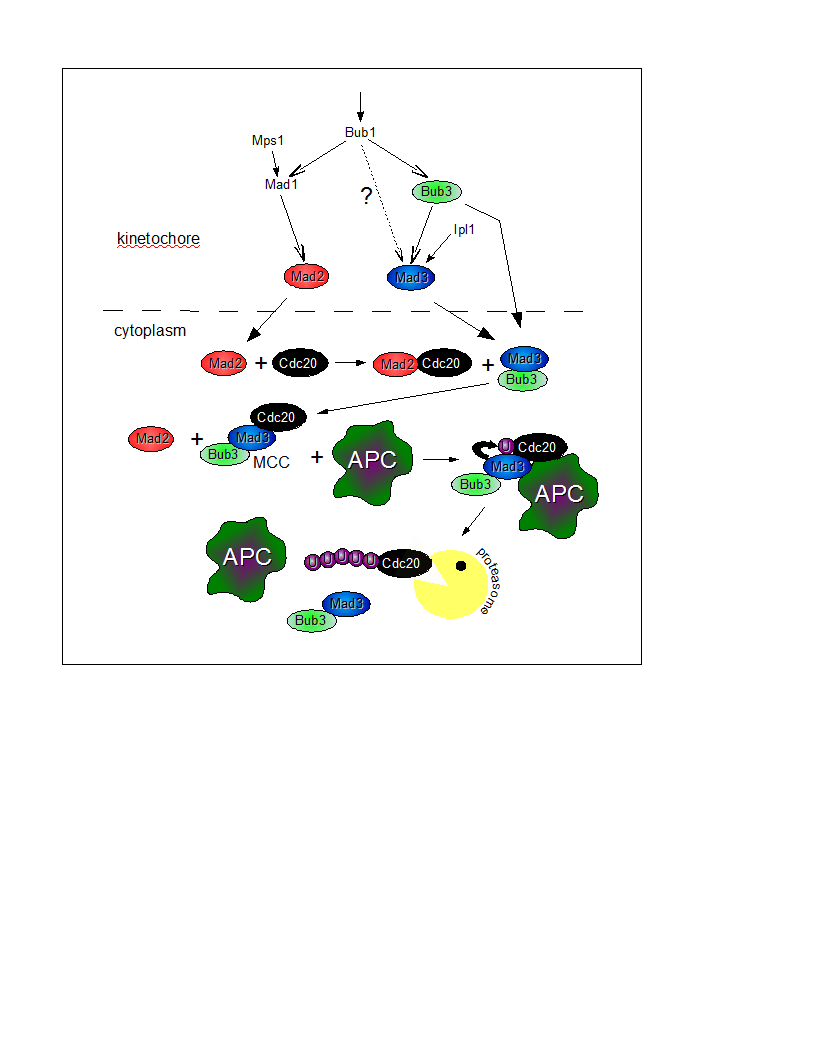


**Figure S14**: It is possible to interpret our results so that the role of Mad2 is limited to shuttling the sequestered Cdc20 to Bub3Mad3(APC) which degrades it.

**10 References**

1. Doncic A, Ben-Jacob E, Barkai N (2005) Evaluating putative mechanisms of the mitotic spindle checkpoint. Proc Natl Acad Sci U S A 102: 6332-6337.

2. Doncic A, Ben-Jacob E, Barkai N (2006) Noise resistance in the spindle assembly checkpoint. Mol Syst Biol 2: 2006 0027.

3. Morris MC, Kaiser P, Rudyak S, Baskerville C, Watson MH, et al. (2003) Cks1-dependent proteasome recruitment and activation of CDC20 transcription in budding yeast. Nature 423: 1009-1013.

4. Poddar A, Stukenberg PT, Burke DJ (2005) Two complexes of spindle checkpoint proteins containing Cdc20 and Mad2 assemble during mitosis independently of the kinetochore in Saccharomyces cerevisiae. Eukaryot Cell 4: 867-878.

5. King EM, van der Sar SJ, Hardwick KG (2007) Mad3 KEN boxes mediate both Cdc20 and Mad3 turnover, and are critical for the spindle checkpoint. PLoS ONE 2: e342.

6. Palframan WJ, Meehl JB, Jaspersen SL, Winey M, Murray AW (2006) Anaphase inactivation of the spindle checkpoint. Science 313: 680-684.

7. Jaspersen SL, Huneycutt BJ, Giddings TH, Jr., Resing KA, Ahn NG, et al. (2004) Cdc28/Cdk1 regulates spindle pole body duplication through phosphorylation of Spc42 and Mps1. Dev Cell 7: 263-274.

8. Schwab M, Lutum AS, Seufert W (1997) Yeast Hct1 is a regulator of Clb2 cyclin proteolysis. Cell 90: 683-693.

9. Cohen-Fix O, Koshland D (1999) Pds1p of budding yeast has dual roles: inhibition of anaphase initiation and regulation of mitotic exit. Genes Dev 13: 1950-1959.

10. Tinker-Kulberg RL, Morgan DO (1999) Pds1 and Esp1 control both anaphase and mitotic exit in normal cells and after DNA damage. Genes Dev 13: 1936-1949.

11. Agarwal R, Cohen-Fix O (2002) Phosphorylation of the mitotic regulator Pds1/securin by Cdc28 is required for efficient nuclear localization of Esp1/separase. Genes Dev 16: 1371-1382.

12. Holt LJ, Krutchinsky AN, Morgan DO (2008) Positive feedback sharpens the anaphase switch. Nature.

13. Lopez-Aviles S, Kapuy O, Novak B, Uhlmann F (2009) Irreversibility of mitotic exit is the consequence of systems-level feedback. Nature 459: 592-595.

14. Warren CD, Brady DM, Johnston RC, Hanna JS, Hardwick KG, et al. (2002) Distinct chromosome segregation roles for spindle checkpoint proteins. Mol Biol Cell 13: 3029-3041.

15. Indjeian VB, Stern BM, Murray AW (2005) The centromeric protein Sgo1 is required to sense lack of tension on mitotic chromosomes. Science 307: 130-133.

16. Fernius J, Hardwick KG (2007) Bub1 kinase targets Sgo1 to ensure efficient chromosome biorientation in budding yeast mitosis. PLoS Genet 3: e213.

17. Hieter P, Mann C, Snyder M, Davis RW (1985) Mitotic stability of yeast chromosomes: a colony color assay that measures nondisjunction and chromosome loss. Cell 40: 381-392.

18. DeAntoni A, Sala V, Musacchio A (2005) Explaining the oligomerization properties of the spindle assembly checkpoint protein Mad2. Philos Trans R Soc Lond B Biol Sci 360: 637-647, discussion 447-638.

19. Hughes TR, Roberts CJ, Dai H, Jones AR, Meyer MR, et al. (2000) Widespread aneuploidy revealed by DNA microarray expression profiling. Nat Genet 25: 333-337.

20. Logarinho E, Resende T, Torres C, Bousbaa H (2008) The human spindle assembly checkpoint protein bub3 is required for the establishment of efficient kinetochore-microtubule attachments. Mol Biol Cell 19: 1798-1813.

21. Kerscher O, Crotti LB, Basrai MA (2003) Recognizing chromosomes in trouble: association of the spindle checkpoint protein Bub3p with altered kinetochores and a unique defective centromere. Mol Cell Biol 23: 6406-6418.

22. Gillett ES, Espelin CW, Sorger PK (2004) Spindle checkpoint proteins and chromosome-microtubule attachment in budding yeast. J Cell Biol 164: 535-546.

23. Pan J, Chen RH (2004) Spindle checkpoint regulates Cdc20p stability in Saccharomyces cerevisiae. Genes Dev 18: 1439-1451.

24. Hardwick KG, Johnston RC, Smith DL, Murray AW (2000) MAD3 encodes a novel component of the spindle checkpoint which interacts with Bub3p, Cdc20p, and Mad2p. J Cell Biol 148: 871-882.

25. Nilsson J, Yekezare M, Minshull J, Pines J (2008) The APC/C maintains the spindle assembly checkpoint by targeting Cdc20 for destruction. Nat Cell Biol 10: 1411-1420.

1. https://portal.biobase-international.com/cgi-bin/portal/login.cgi ). [↑](#footnote-ref-2)
